# Sivelestat sodium alleviates sepsis-associated acute lung injury by inhibiting ferroptosis via the Nrf2/SLC7A11/GPX4 axis

**DOI:** 10.1371/journal.pone.0353525

**Published:** 2026-07-14

**Authors:** Ying Wang, Xiaoling Xia, Yan Lu, ChengLiang Zhang, Yaqing Zhou, Jingjing Fan

**Affiliations:** 1 Department of Critical Care Medicine, Nantong First People’s Hospital, Nantong, Jiangsu, China; 2 Department of Respiratory and Critical Care Medicine, Hai’an Hospital of Traditional Chinese Medicine Affiliated to Nanjing University of Chinese Medicine, Nantong, Jiangsu, China; 3 Department of Obstetrical, Nantong First People’s Hospital, Nantong, Jiangsu, China; 4 Department of Critical Care Medicine, Affiliated Hai’an Hospital of Nantong University, Hai’an County, Nantong, Jiangsu, China; 5 Journal Academic Center, The First Affiliated Hospital with Nanjing Medical University, Nanjing, China; Jinan University, CHINA

## Abstract

**Objective:**

This study was aimed to investigate the therapeutic efficacy and mechanism of Sivelestat sodium in sepsis-associated acute lung injury (SALI).

**Methods:**

A rat model of SALI was established using the cecal ligation and puncture (CLP) procedure. After the model was established in rats, behavioral changes, abdominal conditions, and survival rates were monitored. Comparative analyses included arterial blood gas parameters, the lung wet/dry weight ratio (W/D), lung injury scores, histopathological damage assessment, inflammatory cytokine levels, oxidative stress marker levels, and Fe^2+^ concentrations in lung tissue. The expression levels of Nrf2, SLC7A11, GPX4, ACSL4, and TFR1 proteins in pulmonary tissues were also evaluated.

**Results:**

All rats in the sham group survived. The survival rates in the low-dose (SL), medium-dose (SM), and high-dose (SH) groups (60.0%, 73.3%, and 86.7%, respectively) were higher than those in the CLP group (53.3%), although the differences were not statistically significant. Compared to the Sham group, the CLP group showed significantly lower PaO_2_ and PaO_2_/FiO_2_, higher lung wet/dry weight ratio and lung injury score, higher levels of TNF-α, IL-1β, and IL-6 in the lung tissue, lower levels of SOD and GSH, and higher levels of Fe^2+^ and MDA. Additionally, the expression of Nrf2, SLC7A11, and GPX4 proteins was downregulated, whereas the expression of ACSL4 and TFR1 proteins was upregulated. Compared to the CLP group, the SL, SM, and SH groups showed higher PaO_2_ and PaO_2_/FiO_2_ ratios. The SM and SH groups showed lower lung tissue W/D ratios and lung injury scores, along with lower levels of TNF-α, IL-1β, and IL-6 in the lung tissue. In contrast, only the IL-6 levels were lower in the SL group. The SM and SH groups showed significantly higher SOD and GSH levels, along with considerably lower Fe^2+^ and MDA levels. Notably, the SL group exhibited significant improvement only in arterial blood gas parameters and IL-6 levels, with no statistically significant differences observed in other indicators, indicating a dose-dependent response. In all Sivelestat sodium-treated groups, the expression levels of Nrf2, SLC7A11, and GPX4 proteins in rat lung tissue were upregulated, whereas the expression of ACSL4 and TFR1 proteins was downregulated. To validate the Nrf2 pathway, medium-dose Sivelestat sodium was administered with or without the Nrf2 inhibitor ML385. Sivelestat sodium upregulated total Nrf2, nuclear Nrf2, SLC7A11, and GPX4, and downregulated ACSL4 and TFR1. Co-administration of ML385 largely abolished these changes and blunted its protective effects on lung histopathology.

**Conclusion:**

Sivelestat sodium ameliorates SALI in rats by inhibiting ferroptosis through regulation of the Nrf2/SLC7A11/GPX4 signaling pathway.

## 1 Introduction

Sepsis is a life-threatening organ dysfunction caused by dysregulation of the response of the host’s immune system to infection [1]. Sepsis involves not only systemic inflammatory responses and immune dysregulation but also the effects on multiple organs [2], with the lungs being one of the most commonly affected organs [3]. About 40% of acute lung injuries are associated with sepsis [4]. Sepsis-associated acute lung injury (SALI) results from dual damage to vascular endothelial cells and alveolar epithelial cells, which increases alveolar capillary permeability, decreases surfactant secretion [5], and causes extensive fluid accumulation within the alveoli. These changes decrease residual lung volume and reduce compliance. Its clinical manifestations include progressive refractory hypoxemia and respiratory distress. Treatment techniques for SALI focus mainly on supportive care, with no specific therapeutic drugs or methods available. Rapid disease progression leads to a mortality rate as high as 45% [6]. Although research on SALI has been performed for decades, effective therapeutic targets remain unidentified [7].

Ferroptosis is a novel form of cell death. Morphologically [8], it is characterized by mitochondria dysfunction. Biochemically, it manifests as intracellular iron accumulation, excessive production of reactive oxygen species (ROS), depletion of glutathione (GSH), and increased levels of other mediators, such as arachidonic acid [9]. This leads to the accumulation of lipid peroxides, ultimately inducing cell death. In recent years, several studies have demonstrated that ferroptosis plays a key role in the onset and progression of SALI. In a lipopolysaccharide (LPS)-induced mouse model, the expression of the ferroptosis marker glutathione peroxidase 4 (GPX4) decreased significantly, thereby aggravating LPS-induced SALI. Treatment with ferroptosis inhibitors reversed this effect, indicating that ferroptosis plays an important role in LPS-induced SALI [10]. In a similar mouse model of SALI, it was reported that ginseng extract alleviated pulmonary inflammatory responses and acute lung injury by inhibiting ferroptosis [11]. Therefore, inhibition of ferroptosis represents a novel potential therapeutic strategy for treating SALI.

A comprehensive evaluation of the ferroptosis regulatory mechanism indicates that multiple metabolic pathways are involved in the occurrence of lipid peroxidation. Nuclear factor erythroid 2-related factor 2 (Nrf2) and solute carrier family 7 member 11 (SLC7A11)/GPX4 modulation constitute one of the key endogenous antioxidant defense mechanisms in the body. Ferroptosis plays a crucial role in the treatment of acute lung injury (ALI) by regulating ferroptosis [12]. Therefore, modulation of ferroptosis via the Nrf2/SLC7A11/GPX4 pathway may represent a novel therapeutic approach for SALI.

Sivelestat sodium, a novel therapeutic agent for ALI, can inhibit neutrophil elastase (NE) activity, mitigate neutrophil-mediated inflammatory responses, and has been used in the clinical treatment of ALI. In a retrospective study involving 140 patients with SALI, the Sivelestat sodium group demonstrated a shorter mechanical ventilation duration, reduced ICU length of stay, and lower ICU costs compared to the control group. No adverse events were reported during the study period, confirming the efficacy and safety of Sivelestat sodium in patients with septic ALI [13]. Sivelestat sodium alleviates ALI by improving damage to the alveolar epithelium and vascular endothelium and reversing neutrophil-mediated increases in vascular permeability [14]. Its molecular mechanisms remain to be fully elucidated. In addition to its anti-inflammatory effects, Sivelestat sodium may exert organ-protective effects through other mechanisms. Therefore, in this study, a rat model of SALI was established via CLP and perforation to examine the effects of Sivelestat sodium on SALI in rats and determined the potential underlying molecular mechanisms. We assessed whether Sivelestat sodium alleviates SALI by suppressing ferroptosis through the Nrf2/SLC7A11/GPX4 signaling pathway, thereby providing a basis for the clinical prevention and treatment of SALI.

## 2 Materials and methods

### 2.1 Animals

Male Sprague-Dawley (SD) rats (n = 75; weight: 200 ± 20 g) were provided by the Experimental Animal Center of Nantong University. The animal use permit was SYXK(Su)2023−0029. All rats were housed at the Nantong University Laboratory Animal Center, five per cage, under the following conditions: 12-h/12-h light/dark cycle, room temperature of 25.0 ± 1.0 °C, and relative humidity of 50.0 ± 10.0%. They were acclimated for five days. This study was approved by the Animal Ethics Committee of Nantong University (No. S20251105-002). We respect and treat laboratory animals humanely, uphold animal welfare and ethical standards, and adhere to the “3R” principle. All personnel involved in animal care and experimental operations received specialized training provided by the Experimental Animal Center of Nantong University, and all obtained laboratory animal operation qualifications before the experiment. The modeling procedure was independently performed by a single trained operator to avoid experimental errors caused by inter-operator variability.

### 2.2 Reagents and equipment

Sivelestat sodium was purchased from Shanghai Huilun Jiangsu Pharmaceutical Co., Ltd. The tumor necrosis factor-α (TNF-α), interleukin-1β (IL-1β), and interleukin-6 (IL-6) enzyme-linked immunosorbent assay (ELISA) kits were purchased from Nanjing Jiancheng Bioengineering Institute Co., Ltd (Cat Nos. H052-1-2, H002-1-2, and H007-1-2, respectively). The tissue iron assay kit, total superoxide dismutase (SOD) assay kit, reduced glutathione (GSH) assay kit, and malondialdehyde (MDA) assay kit were purchased from Nanjing Jiancheng Bioengineering Institute Co., Ltd. (Cat Nos. A039-2-1, A001-3-2, A006-2-1, and A003-1-2, respectively). The rabbit anti-Nrf2 antibody (Cat. No. HA723302)was purchased from HUABIO (Cambridge, MA, USA). The rabbit anti-GPX4 antibody (Cat. No. CY6959), rabbit anti-SLC7A11 antibody (Cat. No. CY7046), rabbit anti-transferrin receptor protein 1 (TFR1) antibody (Cat. No. CY6618), rabbit anti-acyl-CoA synthetase long chain family 4 (ACSL4) antibody (Cat. No. DY1198), and rabbit anti-glyceraldehyde phosphate dehydrogenase (GAPDH) antibody (Cat. No. AB0037) were purchased from Shanghai Abways Biotechnology Co., Ltd. The HRP-conjugated goat anti-rabbit IgG (H + L) secondary antibody was purchased from Servicebio (Wuhan, China; Cat. No. G1213). The GEM Premier 5000 Blood Gas Analyzer was purchased from Wofen in the United States, an electronic balance was purchased from Mettler Toledo, Switzerland, a low-temperature refrigerated centrifuge was purchased from Beckman, USA, inverted microscopes and digital imaging equipment were purchased from Olympus Japan, and a microplate reader was purchased from BioTek, USA.

### 2.3 Experimental methods

#### 2.3.1 Modeling and animal grouping.

The male SD rats (n = 75) were randomly divided into five groups: a sham-operated (sham) group, a model group (CLP), and low-dose (SL), medium-dose (SM), and high-dose (SH) Sivelestat sodium groups, with 15 rats in each group.

Model establishment: A sepsis model was established via CLP [15]. The anesthetic method involved intraperitoneal injection of 1% sodium pentobarbital (dose: 60 mg/kg). Preoperative analgesia was provided by a subcutaneous injection of buprenorphine (dose: 0.1 mg/kg) 30 minutes before skin incision and the rats were placed in the supine position. A surgical incision was made in the middle to lower segment of the anterior abdominal midline. The hair around the incision site and within a 2−3 cm radius was shaved using a clipper. Then, the skin was disinfected with povidone-iodine solution twice, followed by one wipe with alcohol to remove the iodine. After disinfection, a 2 cm longitudinal incision was made along the midline of the rat abdomen to expose the cecum. The cecal end was positioned at the midpoint of the ileocecal valve, the mesentery was carefully dissected and ligated with a 4−0 silk suture, and care was taken to avoid damaging the mesenteric vessels. A 7-gauge needle was used to perform a transumbilical puncture at the midpoint of the ligated cecum. Rice-grain-sized cecal contents were gently expelled through the puncture site. Finally, the cecum was repositioned into the abdominal cavity, and the abdominal wall was closed with interrupted sutures. After abdominal closure, 3 mL of saline solution per 100 g of body weight prewarmed at 37 °C was slowly injected subcutaneously to prevent dehydration and septic shock in the rats. In the sham group, the abdomen was immediately closed after the cecum was exposed via laparotomy. All rats were placed on a heating pad after surgery, maintaining a temperature between 36.5 °C and 37.5 °C until fully awake, after which they were returned to their original cages for observation. Postoperative analgesia was maintained with subcutaneous injections of buprenorphine (0.1 mg/kg) every 8 hours for at least 72 hours.

For each Sivelestat sodium intervention group, 2 h after modeling, the following doses of Sivelestat sodium were administered via intraperitoneal injection: 20 mg/kg SL, 40 mg/kg SM, and 80 mg/kg SH. The 40 mg/kg dose is equivalent to 4.8 mg/kg in humans. The 80 mg/kg dose group was designed to evaluate the drug's maximum pharmacodynamic potential and establish a complete dose-response relationship. Doses for each group were set according to the drug instructions and converted to equivalent doses in humans/rats [16]. Sivelestat Sodium powder (100 mg per vial) was dissolved in sterile saline to prepare three working solutions at concentrations of 4, 8, and 16 mg/mL, corresponding to the SL group, SM group, and SH group respectively. Each rat weighing approximately 200 g received an injection of 1.0 mL of the corresponding solution. Sivelestat sodium was administered once every 24 h for three consecutive days. The sham and CLP group received intraperitoneal injections of an equivalent volume (1.0 mL per 200 g rat) of saline solution.

Animals were randomly assigned to experimental groups using a computer-generated random number sequence. All surgical procedures, postoperative care, and data collection were performed by investigators blinded to the group allocation to minimize potential bias.

#### 2.3.2 General situation and survival rate.

After CLP modeling, animals were monitored every 6 h until 72 h post-surgery. The following general conditions of the rats were observed after modeling: activity levels and responses to external stimuli, feeding, respiration and responsiveness during handling. The survival status of the rats was monitored at 6 h, 12 h, 24 h, 36 h, 48 h, 60 h, and 72 h after drug administration, and survival curves were plotted. After rat modeling, euthanasia was performed within 30 minutes if any humane endpoint criteria were met [17], to minimize animal suffering.

#### 2.3.3 Collection and preservation of specimens.

##### 2.3.3.1 Arterial blood gas analysis.

After 72 h of modeling, the rats were anesthetized via intraperitoneal injection of 1% sodium pentobarbital (dose: 60 mg/kg). Then, their necks were exposed, and 2 ML of blood was drawn from the carotid artery. Arterial blood was collected while the rats were spontaneously breathing room air (FiO₂ = 0.21). The arterial blood samples were analyzed using a blood gas analyzer to compare the arterial partial pressure of oxygen (PaO_2_) and oxygenation index (PaO_2_/FiO_2_) among the groups.

##### 2.3.3.2 Pulmonary tissue sampling.

All rats were anesthetized, and the thoracic cavity was opened to expose the heart and lung tissue while the heart was still beating. Normal saline was slowly perfused through the right ventricle to thoroughly flush the pulmonary vasculature until the effluent became clear. Subsequently, the rats were euthanized by cervical dislocation. The right lung was ligated, and tissue from the right upper lobe was removed and gently rinsed with saline solution to determine the W/D ratio of the lung. Half of the left lung tissue from each rat was placed in 4% paraformaldehyde for fixation, followed by dehydration, paraffin embedding, sectioning, and subsequent hematoxylin and eosin (HE) staining. The remaining lung tissue was labeled and stored at －80°C for subsequent analyses.

#### 2.3.4 Lung moisture-dry ratio assessment.

The freshly removed right upper lobe of the rat lung was blotted dry with lint-free filter paper to remove surface fluids and weighed; the wet weight (W) was recorded. Subsequently, the samples were placed in a 70 °C constant-temperature oven for 72 h. Then, the samples were removed and weighed, and the dry weight (D) was recorded. Finally, the wet-to-dry weight ratio (W/D) of lung tissue was calculated to assess the extent of pulmonary edema.

#### 2.3.5 Hematoxylin and eosin staining and lung injury scoring.

Lung tissue was fixed, dehydrated, embedded in paraffin, sectioned, and subjected to HE staining. Pathological features of rat lung tissue were observed under an optical microscope, with two pathologists who were blinded to the experimental groups independently scoring the extent of lung tissue damage [18]. In cases of disagreement, the final score was determined by consensus discussion. For each rat, two sections from the same lung lobe were evaluated. From each section, 10 non-overlapping high-power fields (×400) were randomly selected (20 fields per animal), excluding fields dominated by large airways or vessels. Five parameters were scored on a 0–2 scale as detailed in Table 1. The lung injury score was calculated using the ATS weighting formula, yielding a continuous value from 0 (no injury) to 1 (maximal injury). The final score for each animal was the mean of the two pathologists’ independent assessments.

**Table 1:** Lung Injury Scoring System.

Parameter	Score per field		
	0	1	2
A. Neutrophils in the alveolar space	none	1-5	>5
B. Neutrophils in the interstitial space	none	1-5	>5
C. Hyaline membranes	none	1	>1
D. Proteinaceous debris filling the airspaces	none	1	>1
E. Alveolar septal thickening	<2x	2x-4x	>4x
The resulting lung injury score was calculated as: Score = [(20 × A) + (14 × B) + (7 × C) + (7 × D) + (2 × E)]/(number of fields × 100)			

#### 2.3.6 Inflammatory cytokine assays.

A portion of the lung tissue sample was homogenized using a glass homogenizer on ice and centrifuged (at 3000 × g for 10 min at 4°C) to obtain the supernatant. According to the instructions provided with the kit, the levels of the inflammatory cytokines TNF-α, IL-1β, and IL-6 in lung tissue were measured via ELISA.

#### 2.3.7 Detection of Fe^2+^ and oxidative stress markers.

A portion of the lung tissue sample was homogenized using a glass homogenizer on ice and centrifuged (at 3000 × g for 10 min at 4°C) to obtain the supernatant. The optical density (OD) values were measured using a microplate reader, and the levels of Fe^2+^, SOD, MDA, and GSH in the lung tissue supernatant were subsequently calculated.

#### 2.3.8 Western blotting analysis.

A portion of the lung tissue was placed in an EP tube supplemented with protein lysis buffer to extract total protein, followed by protein quantification and sodium dodecyl sulfate (SDS)-polyacrylamide gel electrophoresis (PAGE). An equal amount of total protein (20 μg) was loaded per lane for electrophoresis. The membrane was blocked with 5% skim milk for 2 h, incubated with primary antibodies (diluted as follows: rabbit anti-Nrf2, 1:2000; rabbit anti-SLC7A11, 1:2000; rabbit anti-GPX4, 1:2000; rabbit anti-ACSL4, 1:2000; rabbit anti-TFR1, 1:2000; rabbit anti-GAPDH, 1:5000) at room temperature for 1 h, and then incubated overnight at 4°C. After the membrane was washed with TBST solution, the HRP-conjugated goat anti-rabbit IgG (H + L) secondary antibodies (diluted at 1:15000) were added, and the mixture was incubated at room temperature for 2 h. GAPDH was used as the internal control. The Odyssey Infrared Imaging System was used to scan and develop the PVDF membrane. Subsequently, the gray values of the target bands were analyzed, the gray values of the target protein were compared with those of GAPDH, and the ratios were calculated as the relative expression levels of the target proteins.

For Western blotting analysis, each lane was loaded with protein extracted from a distinct lung tissue sample obtained from an individual rat. No technical replicates were included in the statistical analysis. Each data point in the quantitative analysis corresponds to a single measurement from one biologically independent animal.

### 2.4 Supplementary mechanistic validation experiment

#### 2.4.1 Reagents and equipment.

The Nrf2 inhibitor ML385 (Cat. No. HY-100523) was purchased from MedChemExpress (USA). The rabbit anti-Lamin B1 antibody was purchased from Wuhan Servicebio Biotechnology Co., Ltd.(Cat Nos. GB111802). The nuclear protein extraction kit (Cat. No. W037-1–1) was purchased from Nanjing Jiancheng Bioengineering Institute Co., Ltd. The transmission electron microscope (Model: HT7800) was purchased from Hitachi (Tokyo, Japan).

#### 2.4.2 Experimental design and animal grouping.

40 male SD rats (200 ± 20 g) were randomly divided into four groups (n = 10): sham, CLP, SM, and SM + ML385. Animal housing, CLP procedures, and sham treatments followed Sections 2.1 and 2.3. All rats received two intraperitoneal injections: at 0 h and 2 h post-CLP. At 0 h, the sham, CLP, and SM groups received vehicle (saline containing 5% DMSO, 1.0 mL/200 g); the SM + ML385 group received ML385 30 mg/kg (6 mg/mL in 5% DMSO/saline). At 2 h, the sham and CLP groups received saline; the SM and SM + ML385 groups received sivelestat sodium 40 mg/kg (8 mg/mL in saline). Rats were sacrificed at 24 h post-CLP for tissue collection.

#### 2.4.3 Hematoxylin and eosin staining and lung injury scoring.

Lung tissues were processed for H&E staining. Histopathological changes were scored by two blinded pathologists according to Sect 2.3.5.

#### 2.4.4 Transmission electron microscopy.

Fresh lung tissue blocks (approximately 1 mm³) were fixed in 2.5% glutaraldehyde, post-fixed in 1% osmium tetroxide, dehydrated, and embedded in epoxy resin. Ultrathin sections were double-stained with uranyl acetate and lead citrate and examined by TEM. Mitochondrial morphology was evaluated for ferroptotic features (shrinkage, increased membrane density, cristae reduction/disappearance). Ten fields per animal were examined and representative images captured.

All TEM images were anonymized for double-blind scoring by two independent researchers according to the Flameng criteria listed in Table 2 [19], with borderline lesions scored as 0.5. At least five intact mitochondria were counted in each microscopic field. Any scoring discrepancies were resolved via consensus discussion. The average Flameng score per sample was calculated for intergroup statistical analysis.

**Table 2:** Flameng Scoring System.

Score	Observation
0	Normal mitochondria (mitochondria appeared highly dense with well-organized intact cristae)
1	Early mild alteration as manifested by slight clearing of matrix density and partial separation of cristae (without obvious swelling or shrinkage)
2	Moderate damage as manifested by aggravated matrix lucency and widespread separation of cristae
3	Typical ferroptotic change: distinct mitochondrial shrinkage, condensed matrix density and extensive disruption of cristae
4	Severe ferroptotic injury with complete loss of cristae, rupture of inner and outer mitochondrial membranes and cytoplasmic vacuolation

#### 2.4.5 Western blotting analysis.

Nuclear protein was extracted using a nuclear protein extraction kit and quantified by BCA assay. Nuclear protein (20 μg/lane) was separated by SDS-PAGE and transferred to PVDF membrane. After blocking (5% non-fat milk, 2 h, room temperature), membranes were incubated with rabbit anti-Nrf2 (1:2000) and rabbit anti-Lamin B1 (1:1000) at room temperature for 1 h then overnight at 4°C. Following TBST washes, HRP-conjugated goat anti-rabbit IgG (1:15000) was applied for 2 h. Lamin B1 served as the nuclear internal control. Bands were visualized by Odyssey system. Each lane represented a distinct animal; no technical replicates were included. Total protein extraction and detection of total Nrf2, SLC7A11, GPX4, ACSL4, TFR1, and GAPDH followed Sect 2.3.8.

### 2.5 Ethics approval

All experimental procedures were approved by the Institutional Animal Care and Use Committee of Nantong University and were in accordance with the ARRIVE guidelines and the guidelines of laboratory rats research at Nantong University.

### 2.6 Statistical analysis

#### 2.6.1 Sample size determination.

Sample size was determined using the resource equation method with an a priori power analysis as confirmation. By the resource equation method, the error degrees of freedom E = 5n − 5 was set to 20, yielding a minimum of 5 rats per group. A priori power analysis using G*Power based on our previously published data [20] indicated a minimum of 3 rats per group (t-test) or 8 rats per group (ANOVA) for the primary outcome (PaO₂/FiO₂ ratio, α = 0.05, power = 0.80). Accounting for expected CLP mortality and adhering to the 3R principles, 15 rats were assigned per group. Post hoc power for the primary outcome exceeded 0.80, confirming adequate effective sample size.

Statistical analysis and graphing were performed using SPSS 26.0 and GraphPad Prism 8.0. The experimental unit was defined as one individual rat. Quantitative data were expressed as the mean ± standard deviation (SD). One-way analysis of variance (ANOVA) was performed followed by Tukey’s post hoc test for multiple comparisons. Categorical data were presented as percentages and analyzed using Chi-square tests. Kaplan-Meier survival curves were constructed, and the log-rank test was used to compare survival distributions among groups. All results were considered statistically significant at P < 0.05.

## 3 Results

### 3.1 General condition and abdominal findings

Postoperative general condition: Rats in the sham group exhibited normal activity levels. Rats in the remaining groups showed progressively increasing behavioral changes 2 h after CLP surgery, including lethargy, erect hair, reduced alertness and activity levels, decreased food and water intake, reduced responsiveness to external stimuli, half-closed eyes, and rapid breathing. As the modeling period progressed, symptoms gradually worsened, leading to death in some cases.

**Fig 1:**
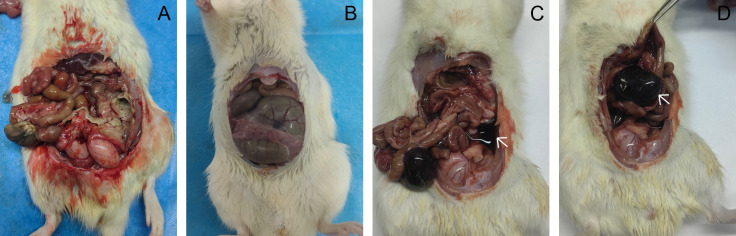
Abdominal Findings. (A) sham, (B) CLP: dilatation of intestine, (C) CLP: abdominal effusion, (D) CLP: intestinal necrosis. Raw data are provided in S1 Data.

The conditions of the abdominal cavity at the time of sampling are shown in Fig 1. In the sham group, no signs of infection, such as bloody exudate or intestinal necrosis, were observed after laparotomy. In contrast, the remaining groups exhibited bloody exudate or purulent discharge after laparotomy, along with abdominal adhesions. The omentum had begun enveloping the ligated portion of the cecum, accompanied by different degrees of intestinal dilatation, edema, and ischemic necrosis. The general postoperative condition and abdominal performance were consistent with those reported in another study on rat models of sepsis [15], indicating successful establishment of the sepsis model.

After Sivelestat sodium was intraperitoneally injected, all groups of rats showed improvements in the aforementioned symptoms, with the SH group showing the most pronounced effect.

### 3.2 Survival conditions

During the observation period, 7, 6, 4, and 2 rats died in the CLP, SL, SM, and SH groups, respectively. Only 1 rat in the CLP group died naturally before meeting the preset humane endpoints; all deaths were primarily due to CLP-induced septic shock.

**Fig 2:**
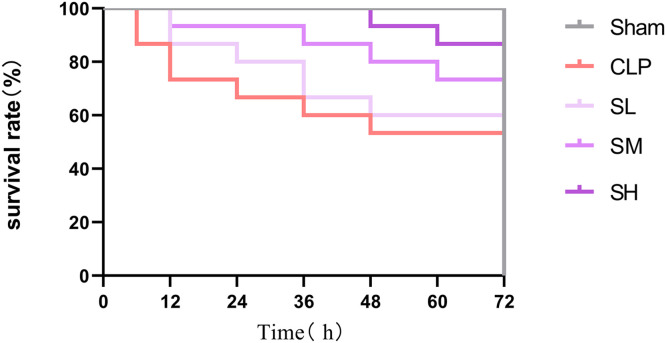
Survival curve at 72 hours post-modeling in rats. Sham group (n = 15, 100% survival). CLP group (n = 15, 53.3% survival). SL, SM, and SH groups (n = 15 per group, survival rates: 60.0%, 73.3%, and 86.7%). No statistically significant differences were found among the groups (*P* = 0.9739;log-rank test). Raw data are provided in S1 Data.

The Kaplan-Meier survival curve is shown in Fig 2. All rats in the sham group survived. The survival rates of rats in the SL, SM, and SH groups (60.0%, 73.3%, and 86.7%, respectively) were higher than those in the CLP group (53.3%), but the differences were not statistically significant (*P* = 0.9739 > 0.05), as determined by the log-rank test.

### 3.3 Analysis of arterial blood gases

**Fig 3:**
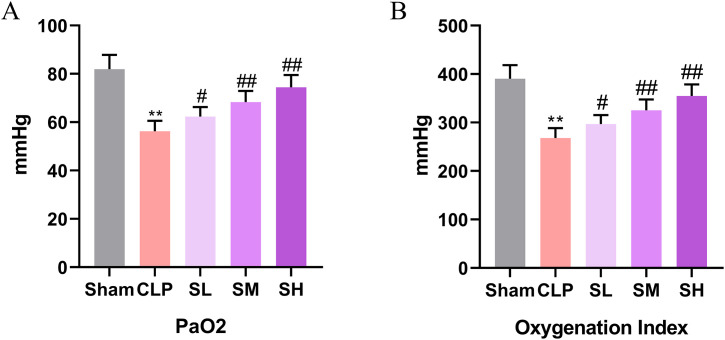
Arterial Blood Gas Analysis. (A) Arterial oxygen partial pressure (PaO₂). (B) Oxygenation index (PaO₂/FiO₂ ratio). Data are presented as mean ± SD. Sample sizes reflect the number of animals that survived to the 72-hour endpoint in each group (n = 15, 8, 9, 11, and 13 for the Sham, CLP, SL, SM, and SH groups, respectively). Statistical significance was determined by one-way ANOVA followed by Tukey’s multiple comparisons test. ^*^P < 0.05,^**^P < 0.01 compared between the Sham and CLP groups; ^#^*P* < 0.05,^##^*P* < 0.01. Compared between the CLP group and the Sivelestat sodium (SL, SM, SH) groups.Raw data are provided in S1 Data.

Compared to those in the sham group, PaO_2_ and the PaO_2_/FiO_2_ ratio were significantly lower in the CLP group (*P* < 0.01) (Fig 3). Compared to those in the CLP group, PaO_2_ and PaO_2_/FiO_2_ were higher in the SL, SM, and SH groups (*P* < 0.05 or 0.01), with the SH group showing a more marked increase. These results indicate that Sivelestat sodium can improve pulmonary oxygenation in SALI rats.

### 3.4 Hematoxylin and eosin staining

**Fig 4:**
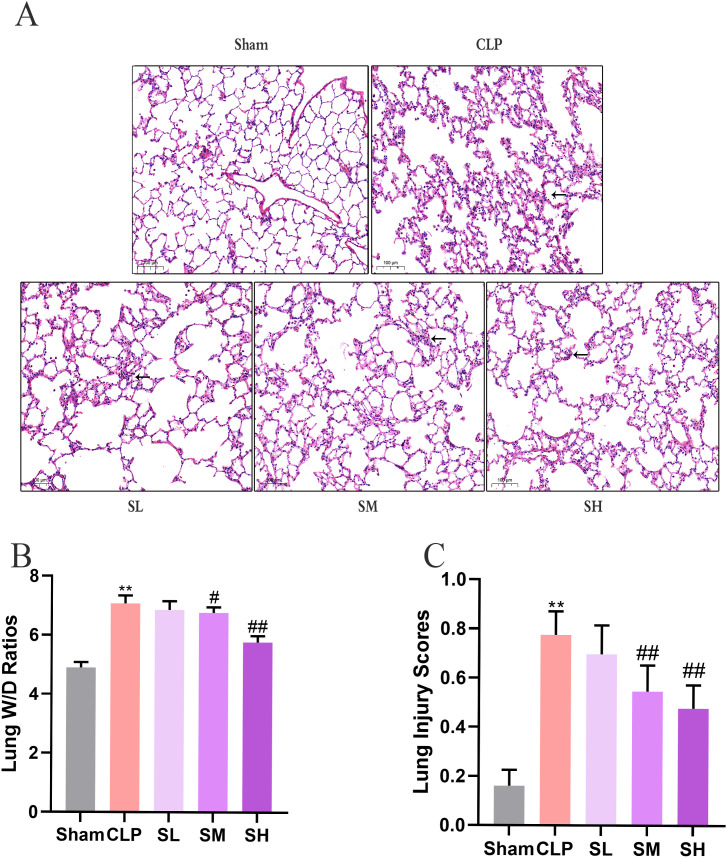
A Comparison of histopathological injury and pulmonary edema among Groups. (A) Representative images of hematoxylin and eosin (H&E)-stained lung sections from each group (Scale bar: 100 µm). (B) Lung tissue water-to-dry (W/D) ratio. (C) Quantitative lung injury score based on histopathological assessment. Data in (B) and (C) are presented as mean ± SD. Sample sizes (n) are as described in the legend of Fig 3. Statistical significance was determined by one-way ANOVA followed by Tukey’s multiple comparisons test. *P < 0.05,**P < 0.01 compared between the Sham and CLP groups; ^#^*P* < 0.05,^##^*P* < 0.01 compared between the CLP group and the Sivelestat sodium (SL, SM, SH) groups. Raw data are provided in S1 Data.

The lung tissue structure of rats in the sham group was intact and well-defined, with no signs of congestion, edema, or inflammatory cell aggregation and no thickening of the pulmonary septa (Fig 4A). The CLP group showed structural disruption of lung tissue, characterized by congestion, edema, inflammatory cell infiltration, partial alveolar collapse, and thickening of the pulmonary septa. After Sivelestat sodium was administered via intraperitoneal injection, the extent of lung tissue damage was reduced, with a certain degree of alleviation of congestion and pulmonary interstitial edema. Focal inflammatory cell infiltration was observed, with marked improvement in the SH group.

### 3.5 Lung tissue W/D ratio and lung injury score

Compared to the Sham group, the CLP group showed significantly higher lung tissue W/D ratios and lung injury scores *(P* < 0.01) (Fig. 4B, 4C). Compared to the CLP group, the SM and SH groups showed significantly lower lung tissue W/D ratios and lung injury scores (*P* < 0.05 or 0.01). These findings suggest that Sivelestat sodium can mitigate sepsis-induced lung injury.

### 3.6 Inflammatory factor levels in lung tissue

**Fig 5:**
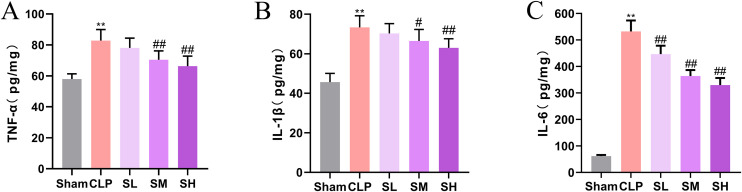
Inflammatory factor levels in lung tissue. (A) Tumor necrosis factor-α (TNF-α). (B) Interleukin-1β (IL-1β), (C) Interleukin-6 (IL-6). Data are presented as mean ± SD. Sample sizes (n) are as described in the legend of Fig 3. Statistical significance was determined by one-way ANOVA followed by Tukey’s multiple comparisons test. *P < 0.05, **P < 0.01 compared between the Sham and CLP groups; ^#^*P* < 0.05, ^##^*P* < 0.01 compared between the CLP group and the Sivelestat sodium (SL, SM, SH) groups. Raw data are provided in S1 Data.

Compared to those in the sham group, the levels of TNF-α, IL-1β, and IL-6 in lung tissue of the CLP group were significantly higher (*P* < 0.01) (Fig 5). Compared to those in the CLP group, the levels of TNF-α, IL-1β, and IL-6 in lung tissues of the SM and SH groups were significantly lower (*P* < 0.05 or 0.01), whereas only the IL-6 level was reduced in lung tissues of the SL group (*P* < 0.01). These results indicate that Sivelestat sodium can reduce inflammatory factor levels in SALI rats, with significant anti-inflammatory effects observed in the medium-dose and high-dose groups.

### 3.7 Levels of Fe^2+^ and oxidative stress markers in lung tissue

**Fig 6:**
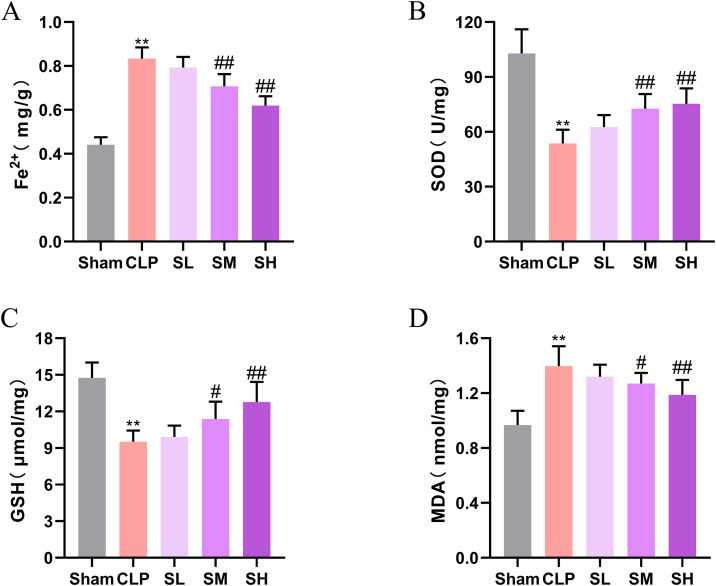
Levels of Fe^2+^ and oxygenation stress markers in lung tissue. (A) Tissue iron (Fe^2+^). (B) Superoxide dismutase (SOD). (C) Glutathione (GSH). (D) Malondialdehyde (MDA). Data are presented as mean ± SD. Sample sizes (n) are as described in the legend of Fig 3. Statistical significance was determined by one-way ANOVA followed by Tukey’s multiple comparisons test. *P < 0.05, **P < 0.01 compared between the Sham and CLP groups; ^#^*P* < 0.05, ^##^*P* < 0.01 compared between the CLP group and the Sivelestat sodium (SL, SM, SH) groups. Raw data are provided in S1 Data.

Compared to those in the Sham group, the levels of SOD and GSH in lung tissue of the CLP group were significantly lower, whereas the levels of Fe^2+^ and MDA were significantly higher (*P* < 0.01) (Fig 6). Compared to those in the CLP group, the levels of SOD and GSH in the lung tissue of the SM and SH groups were significantly higher (*P* < 0.05 or 0.01), whereas the levels of Fe^2+^ and MDA were significantly lower (*P* < 0.05 or 0.01). These findings indicate that Sivelestat sodium reduces the accumulation of iron ions and lipid peroxidation products in lung tissue of SALI rats while increasing the activity of the antioxidants SOD and GSH, thereby mitigating oxidative damage.

### 3.8 Protein expression of Nrf2, SLC7A11, GPX4, ACSL4, and TFR1 in lung tissue

**Fig 7:**
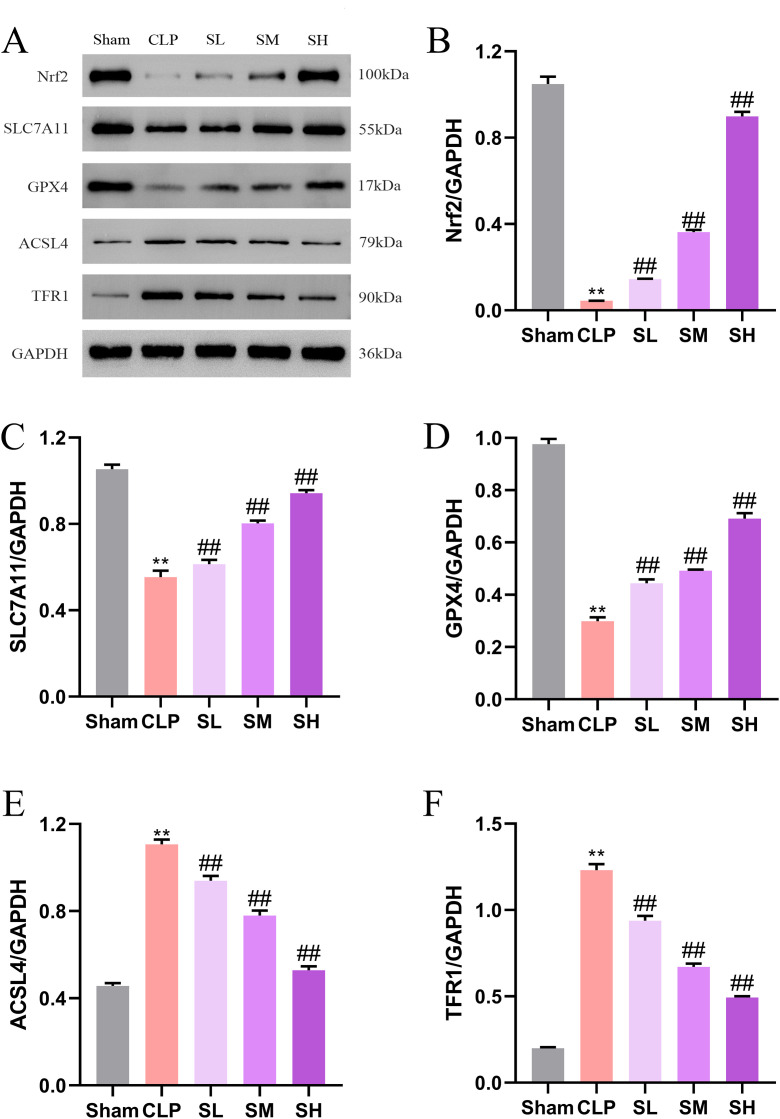
Expression of ferroptosis-Related Proteins in Lung Tissue. (A) Representative Western blot images showing the protein levels of Nuclear factor erythroid 2-related factor 2 (Nrf2), Solute carrier family 7 member 11 (SLC7A11), Glutathione peroxidase 4 (GPX4), Acyl-CoA synthetase long chain family 4 (ACSL4), and Transferrin receptor protein 1 (TFR1). (B-F) Quantitative analysis of the relative expression levels of (B) Nrf2, (C) SLC7A11, (D) GPX4, (E) ACSL4, and (F) TFR1. Data are presented as mean ± SD. Sample sizes (n) are as described in the legend of Fig 3. Statistical significance was determined by one-way ANOVA followed by Tukey’s multiple comparisons test. *P < 0.05, **P < 0.01 compared between the Sham and CLP groups; ^#^*P* < 0.05, ^##^*P* < 0.01 compared between the CLP group and the Sivelestat sodium (SL, SM, SH) groups. Raw data are provided in S1 Data. Uncropped blot images are provided in S2 Raw Images.

Compared to the sham group, the CLP group showed significantly lower expression of Nrf2, SLC7A11, and GPX4 proteins (*P* < 0.01), whereas the expression of ACSL4 and TFR1 proteins was significantly higher (*P* < 0.01) (Fig 7). Compared to those in the CLP group, the expression levels of Nrf2, SLC7A11, and GPX4 proteins were significantly higher (*P* < 0.01) in rat lung tissue across all doses of Sivelestat sodium, whereas ACSL4 and TFR1 protein expression was significantly lower (*P* < 0.01). Sivelestat sodium upregulated the key ferroptosis-related pathway Nrf2/SLC7A11/GPX4, downregulated the expression of ACSL4 and TFR1, inhibited ferroptosis, reduced oxidative damage, and thereby exerted a protective effect in the SALI model.

## 4 Nrf2 inhibition abolishes the protective effect of sivelestat sodium

To determine whether the protective effect of sivelestat sodium is Nrf2-dependent, a supplementary experiment was conducted using the medium dose (SM, 40 mg/kg) and the Nrf2 inhibitor ML385. During the observation period, 2, 1, and 2 rats died in the CLP, SM, and SM + ML385 groups, respectively, all due to CLP-induced septic shock; no deaths occurred before humane endpoints were reached.Because tissue analyses were confined to animals that survived to the 24-h endpoint, these results are also subject to survivorship bias.As a CLP + ML385 control was not included, an inhibitor-independent effect of ML385 cannot be formally excluded.

### 4.1 Hematoxylin and eosin staining

**Fig 8:**
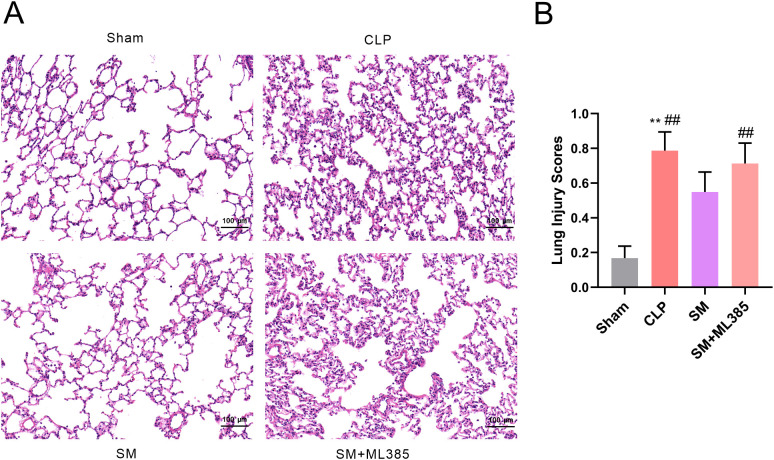
Comparison of histopathological injury. (A) Representative images of hematoxylin and eosin (H&E)-stained lung sections from each group (Scale bar: 100 µm). (B) Quantitative lung injury score based on histopathological assessment. Data is presented as mean ± SD. Sample sizes reflect the number of animals that survived to the 24-hour endpoint in each group (n = 10, 8, 9, and 8 for the Sham, CLP, SM, and SM + ML385 groups, respectively). Statistical significance was determined by one-way ANOVA followed by Tukey’s multiple comparisons test. *P < 0.05,**P < 0.01 compared between the Sham and CLP groups; ^#^*P* < 0.05,^##^*P* < 0.01 compared with the SM group. Raw data are provided in S1 Data.

The sham group showed intact lung structure with no congestion, edema, or inflammatory cell infiltration (Fig 8A). The CLP group exhibited severe lung injury, including congestion, edema, inflammatory cell infiltration, alveolar collapse, and septal thickening. Sivelestat sodium treatment significantly reduced these pathological changes, with only focal inflammation remaining. In the SM + ML385 group, the protective effect was partially reversed, and the injury severity was intermediate between the CLP and SM group.

Compared to the Sham group, the CLP group showed a significantly higher lung injury score (*P* < 0.01) (Fig 8B). The SM group had a significantly lower score than the CLP group (*P* < 0.01),Direct comparison between the SM and SM + ML385 groups confirmed that the lung injury score was significantly higher in the SM + ML385 group (*P* < 0.01), indicating that ML385 largely reversed the protective effect of sivelestat sodium.

### 4.2 Transmission electron microscopy of mitochondrial ultrastructure

**Fig 9:**
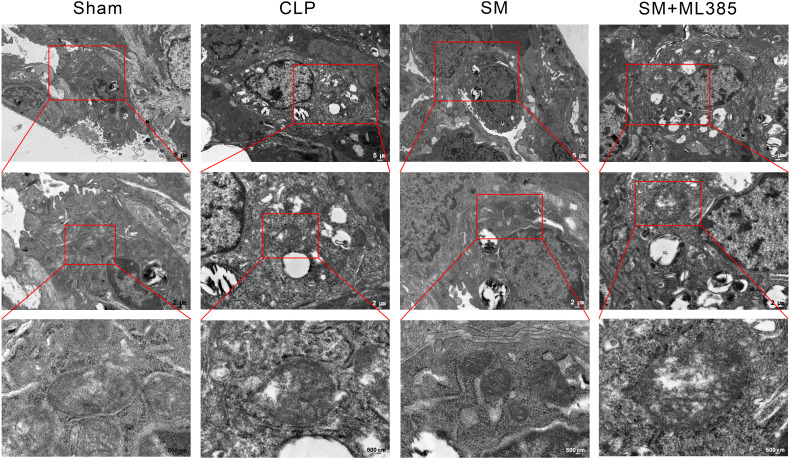
Comparison of mitochondrial ultrastructure in lung tissues (transmission electron microscopy,scale bars: 5 µm, 2 µm, 500 nm). Raw data are provided in S1 Data.

Transmission electron microscopy revealed that the CLP group exhibited typical ferroptosis features: mitochondrial shrinkage, extensive cristae reduction, and increased membrane density, confirming the presence of ferroptosis in the model (Fig 9). The SM group restored mitochondrial morphology with intact cristae and dense matrix, indicating that Sivelestat sodium effectively attenuates ferroptosis-induced mitochondrial damage. However, the SM + Nrf2 inhibitor group showed severe mitochondrial destruction (cristae loss, shrinkage, vacuolation) comparable to the CLP group, indicating that the Nrf2 inhibitor can reverse the protective effect of SM. These findings demonstrate that SM inhibits ferroptosis via the Nrf2 pathway.

**Fig 10:**
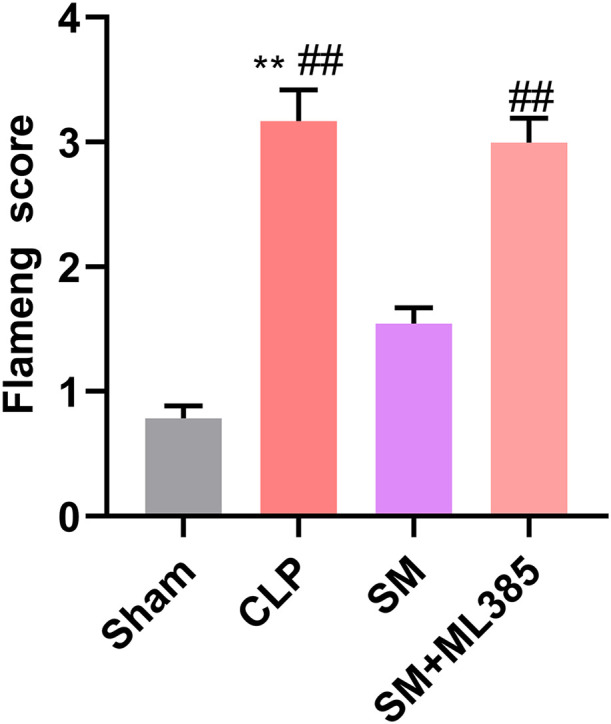
Mitochondrial injury scores in lung tissues. Data is presented as mean ± SD. Sample sizes reflect the number of animals that survived to the 24-hour endpoint in each group (n = 10, 8, 9, and 8 for the Sham, CLP, SM, and SM + ML385 groups, respectively). Statistical significance was determined by one-way ANOVA followed by Tukey’s multiple comparisons test. *P < 0.05,**P < 0.01 compared between the Sham and CLP groups; ^#^*P* < 0.05,^##^*P* < 0.01 compared with the SM group.Raw data are provided in S1 Data.

Compared to the Sham group, the CLP group showed a significantly higher Flameng score (*P* < 0.01) (Fig 10), indicating severe mitochondrial injury. The SM group had a significantly lower score than the CLP group (*P* < 0.01), suggesting that sivelestat sodium attenuates CLP-induced mitochondrial damage. Direct comparison between the SM and SM + ML385 groups confirmed that the Flameng score was significantly higher in the SM + ML385 group (*P* < 0.01), indicating that ML385 largely reversed the protective effect of sivelestat sodium on mitochondrial morphology.

### 4.3 Protein expression in lung tissue

**Fig 11:**
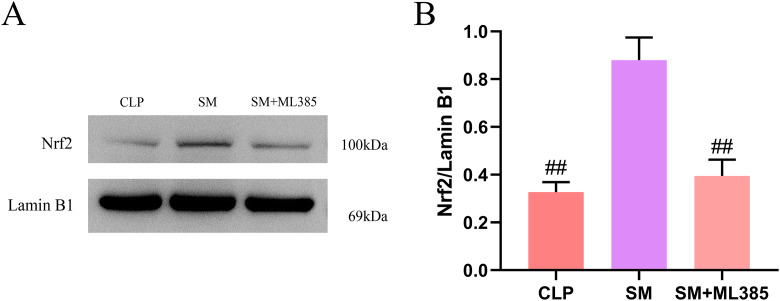
Nuclear Nrf2 protein expression in lung tissues. (A) Representative Western blot of nuclear Nrf2 protein, with Lamin B1 as the nuclear loading control. (B) Quantification of nuclear Nrf2 expression. Data are presented as mean ± SD. Sample sizes reflect the number of animals that survived to the 24-hour endpoint in each group (n = 8, 9,and 8 for the CLP, SM, and SM + ML385 groups, respectively). Statistical significance was determined by one-way ANOVA followed by Tukey’s multiple comparisons test. ^#^*P* < 0.05,^##^*P* < 0.01 compared with the SM group. Raw data are provided in S1 Data. Uncropped blot images are provided in S2 Raw Images.

Nuclear Nrf2 protein expression in lung tissues was examined by Western blotting. The SM group exhibited higher nuclear Nrf2 levels compared to the CLP group (*P* < 0.01) (Fig 11), indicating that Sivelestat sodium promotes Nrf2 nuclear translocation. Nuclear Nrf2 expression in the SM + ML385 group was significantly lower than that in the SM group (*P* < 0.01), confirming that ML385 blocks SM-induced Nrf2 nuclear accumulation.

**Fig 12:**
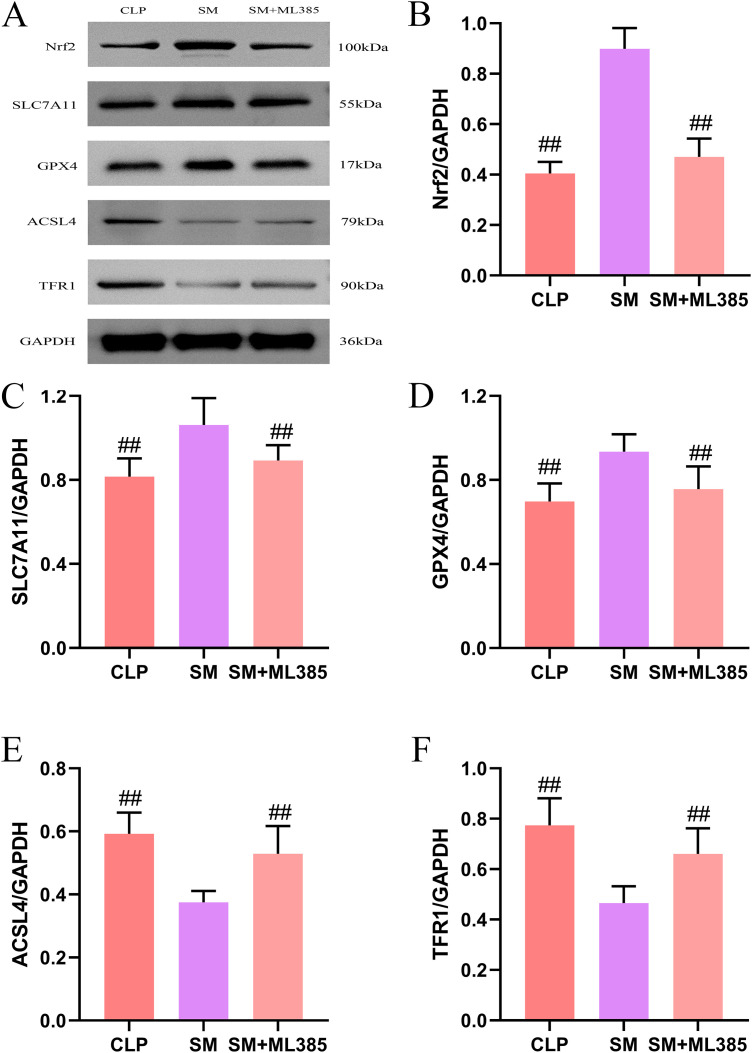
Sivelestat sodium regulates ferroptosis-related proteins via Nrf2. (A) Representative Western blot images showing the protein levels of Nrf2, SLC7A11, GPX4, ACSL4, and TFR1. (B-F) Quantitative analysis of the relative expression levels of (B) Nrf2, (C) SLC7A11, (D) GPX4, (E) ACSL4, and (F) TFR1.Data are presented as mean ± SD. Sample sizes reflect the number of animals that survived to the 24-hour endpoint in each group (n = 8, 9, and 8 for the CLP, SM, and SM + ML385 groups, respectively). Statistical significance was determined by one-way ANOVA followed by Tukey’s multiple comparisons test. ^#^*P* < 0.05,^##^*P* < 0.01 compared with the SM group. Raw data are provided in S1 Data. Uncropped blot images are provided in S2 Raw Images.

The expression of Nrf2, GPX4, SLC7A11, ACSL4, and TFR1 in lung tissues was examined by Western blotting (Fig 12). Compared with the CLP group, the SM group exhibited significantly upregulated Nrf2, GPX4, and SLC7A11 and downregulated ACSL4 and TFR1 (all *P* < 0.01), Direct comparisons between the SM and SM + ML385 groups revealed significant differences in the levels of all these proteins (all *P* < 0.01), confirming that Sivelestat sodium regulates ferroptosis-related proteins in an Nrf2-dependent manner.

## 5 Discussion

Sepsis is an extremely complex and life-threatening clinical syndrome. Each year, more than 30 million people are diagnosed with sepsis, resulting in five million deaths. Survivors often experience long-term consequences [21]. SALI is one of the earliest and most common complications in patients with sepsis and is an independent risk factor for poor prognosis in these patients [22]. Further clarification of the pathogenesis of SALI and identification of safe and effective drugs and treatment methods are urgently needed. The CLP model mimics sepsis caused by clinical gastrointestinal perforation. Compared to exogenous administration of LPS, it induces inflammatory, hemodynamic, and biochemical changes that more closely resemble those observed in human sepsis and is considered the “gold standard” for sepsis models [23]. In this study, the CLP method was used to establish a rat model of sepsis. After surgery, rats exhibited different degrees of behavioral changes, including lethargy, dyspnea, erect hair, decreased alertness and activity levels, reduced food and water intake, and decreased responsiveness to external stimuli. After laparotomy, bloody exudate or purulent fluid was observed in the abdominal cavity, accompanied by different degrees of intestinal dilation, edema, and ischemic necrosis. The CLP group exhibited a survival rate of only 53.3% at 72 h after surgery. The histopathological symptoms of lung tissue included destruction of lung structure, alveolar congestion and edema, accumulation of large numbers of inflammatory cells, alveolar collapse, and thickening of the pulmonary septa. Additionally, the lung tissue wet-dry weight ratio and lung injury score increased significantly. Arterial blood gas analysis showed significantly reduced PaO₂ and PaO₂/FiO₂ ratios in the CLP group, with an oxygenation index of only 267.86 ± 20.6 mmHg. According to the American Thoracic Society (ATS) diagnostic criteria for experimental acute lung injury in animal models [24], the CLP group in the present study exhibited histological evidence of tissue injury (including alveolar congestion, edema, and inflammatory cell infiltration), an inflammatory response (significantly elevated levels of TNF-α, IL-1β, and IL-6), and evidence of physiological dysfunction (significantly reduced PaO₂ and PaO₂/FiO₂ ratios). Collectively, these findings meet the established criteria for a successful SALI model. Several studies have demonstrated that ferroptosis plays a crucial role in the pathogenesis of SALI [25], and inhibiting ferroptosis has been shown to effectively ameliorate sepsis-induced acute lung injury [26]. In this study, Sivelestat sodium significantly improved lung injury and oxygenation in SALI rats, alleviating pulmonary inflammation and damage caused by oxidative stress. This protective effect of Sivelestat sodium may be closely associated with the activation of the Nrf2 pathway, which subsequently increases the expression of its downstream key antiferroptosis proteins SLC7A11 and GPX4.

### 5.1 Protective effects of Sivelestat sodium in SALI

A multicenter, double-blind, randomized controlled trial in China revealed that the application of Sivelestat sodium in patients with sepsis can suppress inflammatory response, prevent the occurrence of SALI, and reduce mortality in sepsis patients [27]. Our study also showed that intraperitoneal administration of Sivelestat sodium improved the general condition and intraperitoneal infection status of rats after CLP surgery. This resulted in improved pulmonary tissue pathological damage and scoring. Compared to those in the CLP group, PaO_2_ and PaO_2_/FiO_2_ levels increased across all dose groups, with the SH group showing a more marked increase, confirming the significant protective effect of Sivelestat sodium against SALI in rats.

Sepsis is one of the most common risk factors for ALI. NE is considered a key mediator in ALI and is extensively involved in multiple processes, including disruption of the alveolar-capillary barrier, oxidative stress and inflammatory responses, autophagy, and cell death [28]. In both in vivo and in vitro studies of the SALI model, Sivelestat sodium, acting as an NE inhibitor, reduces the levels of TNF-α, IL-1β, and IL-6, thereby suppressing systemic inflammatory responses [20]. It inhibits the transforming growth factor-β (TGF-β) signaling pathway, consequently alleviating SALI [29]. Our results are consistent with these findings, as medium and high doses of Sivelestat sodium significantly reduced the levels of TNF-α, IL-1β, and IL-6 in lung tissue. The protective effect of Sivelestat sodium on the lungs is associated with its significant anti-inflammatory action. Some studies have investigated the potential mechanisms of Sivelestat sodium in SALI and found that Sivelestat sodium not only effectively reduces inflammatory responses but also mitigates oxidative stress-induced damage. The application of Sivelestat sodium reduces the levels of inflammatory cytokine levels and intracellular MDA content, increases the levels of SOD and GSH in lung tissue, and alleviates pathological damage [30]. We obtained similar results in our study. Compared to those in the CLP group, the levels of SOD and GSH in the lung tissue were higher, whereas the MDA and Fe² ⁺ levels were lower in the SM and SH groups. These findings suggest that Sivelestat sodium increases the activity of antioxidants SOD and GSH, thereby mitigating oxidative damage. It was hypothesized that Sivelestat sodium may exert its effects not only through direct anti-inflammatory mechanisms but also by enhancing its cellular antioxidant capacity.

In the present study, the dose groups were established with distinct pharmacological rationales. The SM group received 40 mg/kg (equivalent to 4.8 mg/kg in humans), which corresponds to the clinically recommended dosage specified in the drug prescribing information. The SL and SH groups were included to evaluate the maximal therapeutic potential of sivelestat sodium and to establish a comprehensive dose-response relationship.

Our results demonstrated that the SL group exhibited significant improvements in arterial blood gas parameters and IL-6 levels. However, no statistically significant differences were observed in other key indicators, including the lung wet/dry weight ratio, lung injury score, TNF-α, IL-1β, Fe^2+^and oxidative stress-related parameters. We hypothesize,though this remains speculative,that this selective pattern of improvement reflects a threshold-dependent dose–response relationship. Specifically, lower doses of sivelestat sodium appear sufficient to improve early, mild, and rapidly responsive indices, such as the oxygenation index and the acute pro-inflammatory cytokine IL-6 [31]. In contrast, higher drug doses are required to counteract the severe oxidative stress and histopathological damage induced by sepsis. Collectively, these findings indicate that, in the context of SALI, the medium dose (40 mg/kg, SM group) represents the critical effective dosage required for sivelestat sodium to fully exert its pulmonary protective effects.

### 5.2 Sivelestat sodium plays a lung-protective role by inhibiting ferroptosis through the Nrf2/SLC7A11/GPX4 axis

Ferroptosis is a distinct form of programmed cell death that differs from other types of cell death, such as necrosis, apoptosis, autophagy, and pyroptosis. Its most characteristic biological hallmark is the abnormal intracellular accumulation of iron-dependent lipid peroxidation products [32]. Ferroptosis is also associated with LPS-induced ALI [33], and significant progress has been made in studying its regulatory mechanisms [34]. Nrf2 is a key negative regulator of ALI-associated ferroptosis. Activated Nrf2 induces the expression of SLC7A11, promotes GSH synthesis, activates the expression of GPX4, and inhibits ferroptosis-related lipid peroxidation [35]. Moreover, multiple genes involved in iron metabolism and lipid peroxidation are transcriptionally regulated by Nrf2. Blocking Nrf2 degradation and increasing Nrf2 levels can promote the transcription of the target genes SLC7A11/GPX4, thereby suppressing the occurrence of ferroptosis [36]. Therefore, the Nrf2/SLC7A11/GPX4 signaling pathway is a critical pathway regulating ferroptosis. ACSL4 is a lipid metabolism enzyme recognized as a specific biomarker and driver of ferroptosis [37]. ACSL4 promotes ferroptosis by facilitating the esterification of polyunsaturated fatty acids into acyl-CoA. The uptake of cellular iron is regulated primarily by plasma membrane protein transferrin receptor 1 (TFR1). This protein facilitates the endocytosis of transferrin-bound iron into cells. Therefore, TFR1 is considered a marker protein for ferroptosis. Knocking out TFR1 to block this process prevents ferroptosis [38]. Thus, ACSL4 and TFR1 are important pharmacological targets for treating ferroptosis-related diseases. In our study, iron deposition and oxidative stress damage were observed in the lung tissue of the CLP group, accompanied by alterations in marker protein expression. The expression of Nrf2, SLC7A11, and GPX4 proteins was downregulated, whereas the expression of ACSL4 and TFR1 proteins was upregulated, indicating the occurrence of ferroptosis in SALI.

Ferroptosis inhibitors can scavenge oxygen free radicals, inhibit the downregulation of the ferroptosis negative markers SLC7A11 and GPX4, mitigate LPS-induced lung tissue injury, alleviate pulmonary edema, and reduce pulmonary vascular permeability. These findings indicate that SALI is associated with ferroptosis and that simultaneously inhibiting ferroptosis alleviates lung injury [39]. In this study, following the intraperitoneal administration of Sivelestat sodium, Fe^2+^ levels decreased in rat lung tissue, oxidative damage decreased, and the Nrf2/SLC7A11/GPX4 pathway was upregulated, thereby increasing the cellular antioxidant defense capacity. Simultaneously, the downregulation of ACSL4/TFR1 inhibited lipid peroxidation and iron uptake. These results confirm the lung-protective effect of Sivelestat sodium and suggest that it may be achieved by inhibiting ferroptosis through the Nrf2/SLC7A11/GPX4 axis.

To determine whether these protective effects depend on Nrf2 activation, medium-dose Sivelestat sodium was combined with the Nrf2 inhibitor ML385. ML385 largely abolished Sivelestat-induced upregulation of total Nrf2, nuclear Nrf2, SLC7A11, and GPX4, restored ACSL4 and TFR1 expression, and attenuated its protective effects on lung histopathology. These findings indicate that the lung-protective action of Sivelestat sodium is Nrf2-dependent.

This study not only confirms the anti-inflammatory properties of Sivelestat sodium in SALI but also unveils a mechanism through which it attenuates ferroptosis and oxidative damage by activating the Nrf2/SLC7A11/GPX4 pathway. These insights broaden the understanding of Sivelestat sodium’s mechanism of action and provide a theoretical basis for its potential application as a multi-target agent in SALI treatment. However, this study has several limitations that should be considered when interpreting the findings. First and most importantly, all tissue-level analyses (including histology, inflammatory markers, and protein expression) were performed exclusively on animals that survived to the 72-hour endpoint. Animals that died prior to this time point were excluded from these analyses. This introduces a significant survivorship bias, as the data represent only the subset of rats that were able to survive the septic insult. Consequently, the observed therapeutic benefits of Sivelestat sodium in pulmonary pathology and molecular markers may be overestimated. Second, the interpretation of survival outcomes is further constrained by the limited sample size. Although a dose-dependent trend toward increased survival was observed, the lack of statistical significance (*P* = 0.9739) may reflect insufficient power to detect a true difference in mortality given the modest group sizes. Third, the mechanistic validation experiment was conducted at 24 h post-CLP with single-dose Sivelestat sodium (40 mg/kg), whereas the main efficacy study employed a 72-h endpoint with repeated dosing. Although the consistent 40 mg/kg dose provides a pharmacological link, Nrf2-dependence of ferroptosis inhibition at 72 h is inferred from these acute data and requires confirmation in future studies.Future studies should incorporate predefined interim sampling time points to capture data from non-surviving animals and utilize larger sample sizes to more robustly assess the impact of Sivelestat sodium on survival.Additionally, a standalone CLP + ML385 control group should be included in follow-up experiments to rule out confounding effects of ML385 per se on lung injury and ferroptosis-related markers, thereby strengthening the causal evidence that sivelestat sodium alleviates lung damage through Nrf2-dependent signaling.

## Supporting information

S1 Data Raw data of all experimental results presented in this study. (XLSX)

S2 Raw Images Uncropped original blots/gels corresponding to Figure 7A, Figure 11A, and Figure 12A. (PDF)
