# Supplementary material for: Sivelestat sodium alleviates sepsis-associated acute lung injury by inhibiting ferroptosis via the Nrf2/SLC7A11/GPX4 axis
Source: PLoS One. 2026 Jul 14;21(7):e0353525. doi: 10.1371/journal.pone.0353525 (PMC13367734; doi:10.1371/journal.pone.0353525)
Supplement: S2 Raw Images — (PDF) [file pone.0353525.s002.pdf]

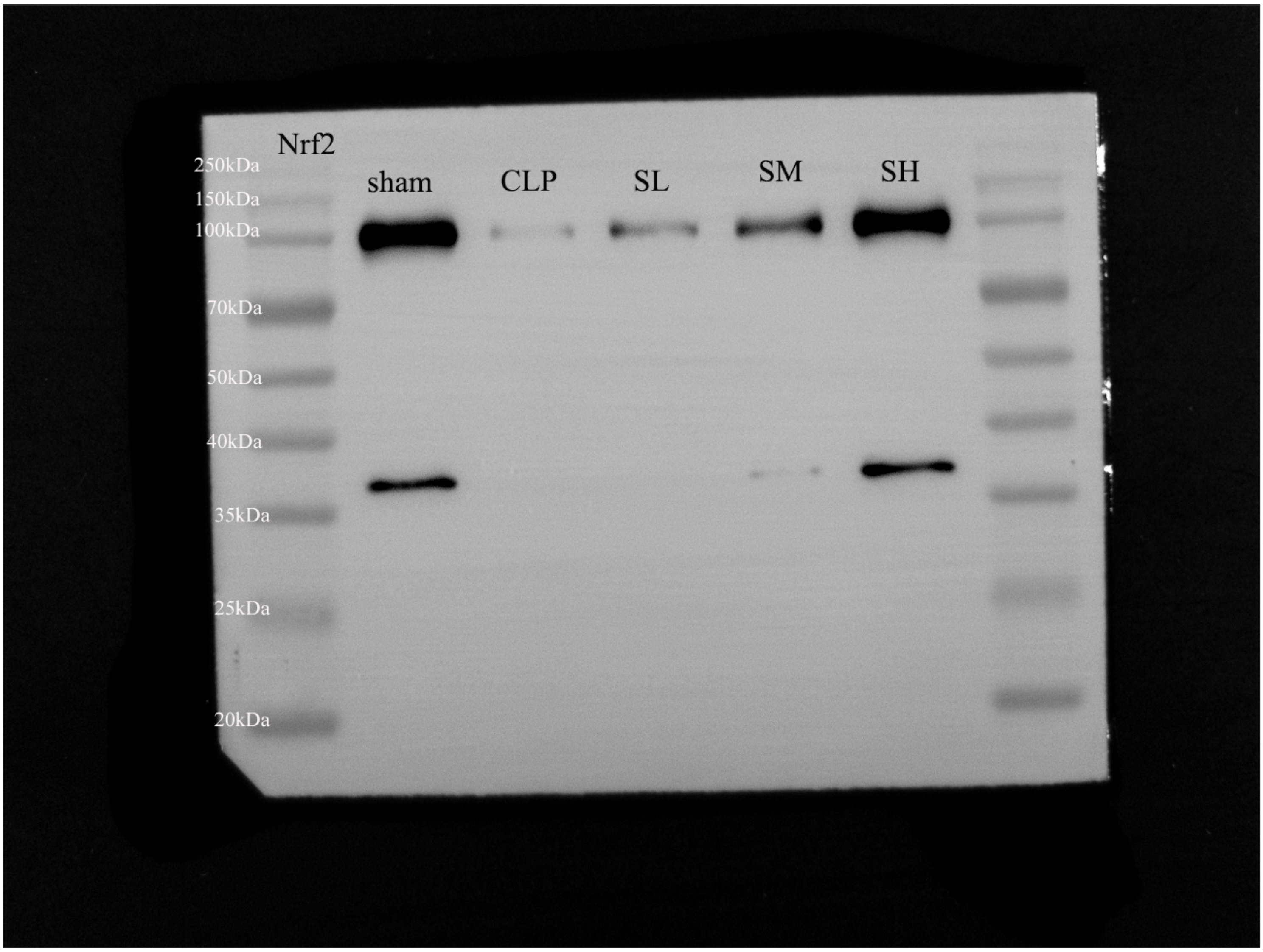


Representative Western blot images of NRF2 protein expression in lung tissue.Fig 7 was generated from this original image.

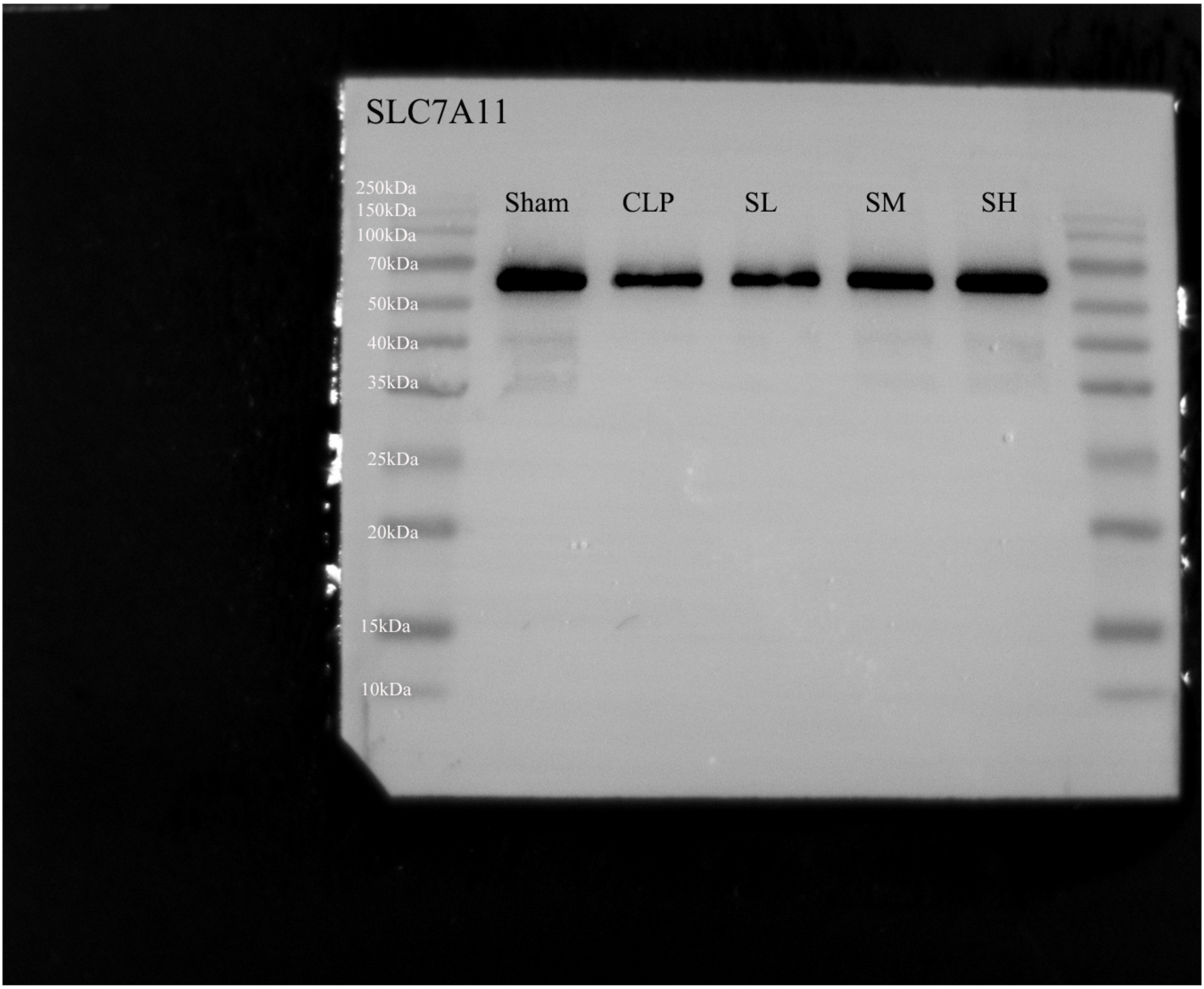


Representative Western blot images of SLC7A11 protein expression in lung tissue.Fig 7 was generated from this original image.

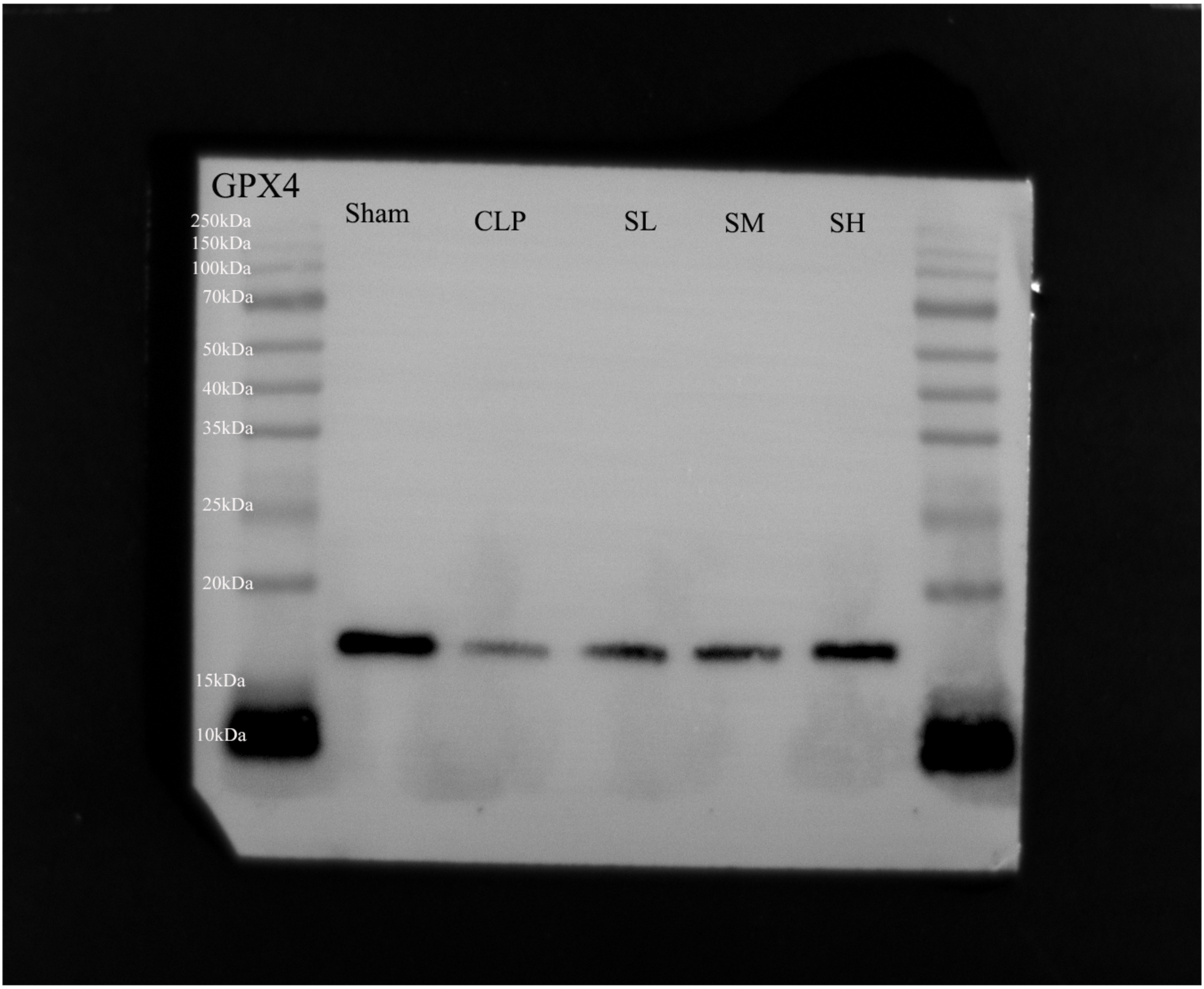


Representative Western blot images of GPX4 protein expression in lung tissue.Fig 7 was generated from this original image.

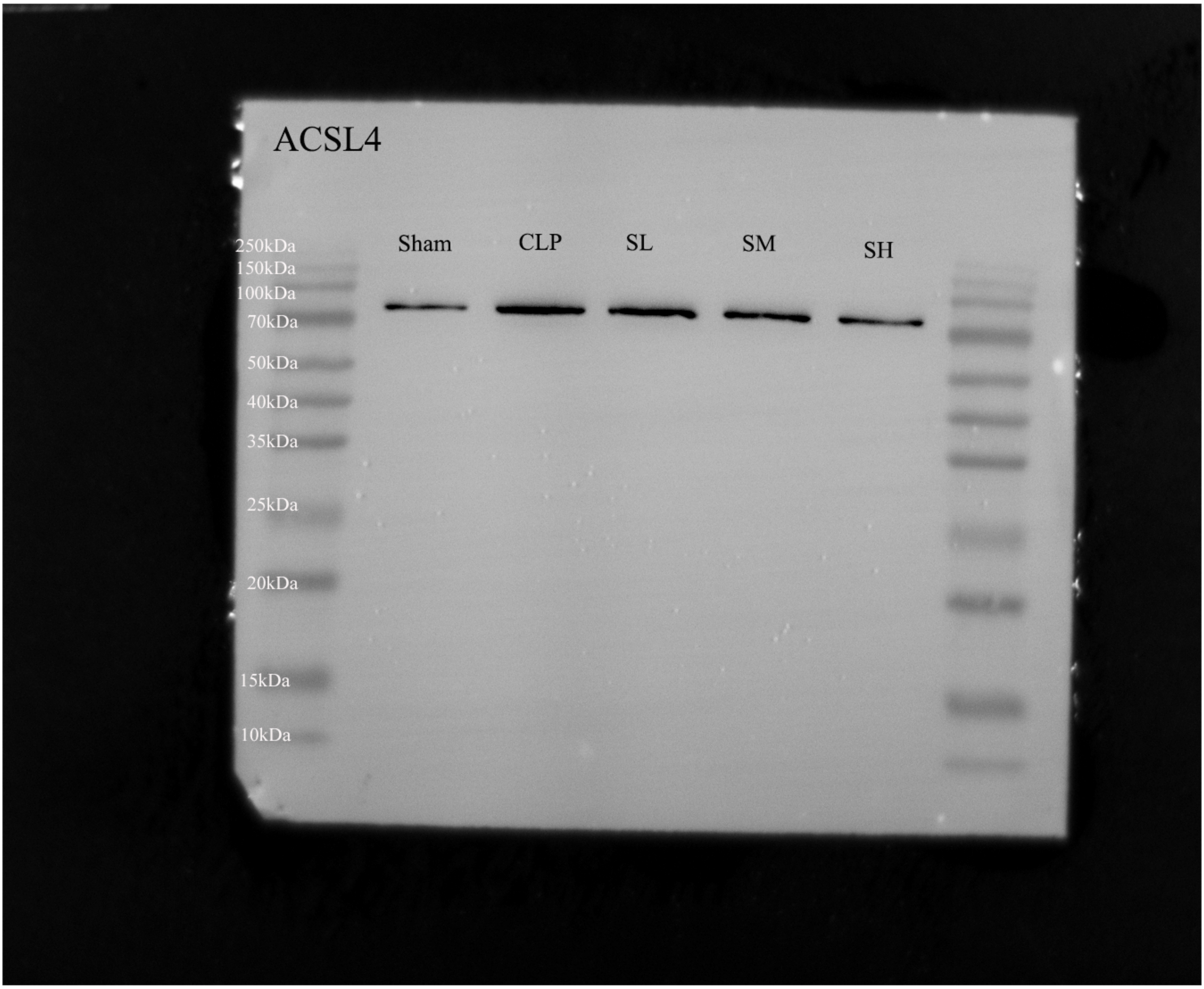


Representative Western blot images of ACSL4 protein expression in lung tissue. Fig 7 was generated from this original image.

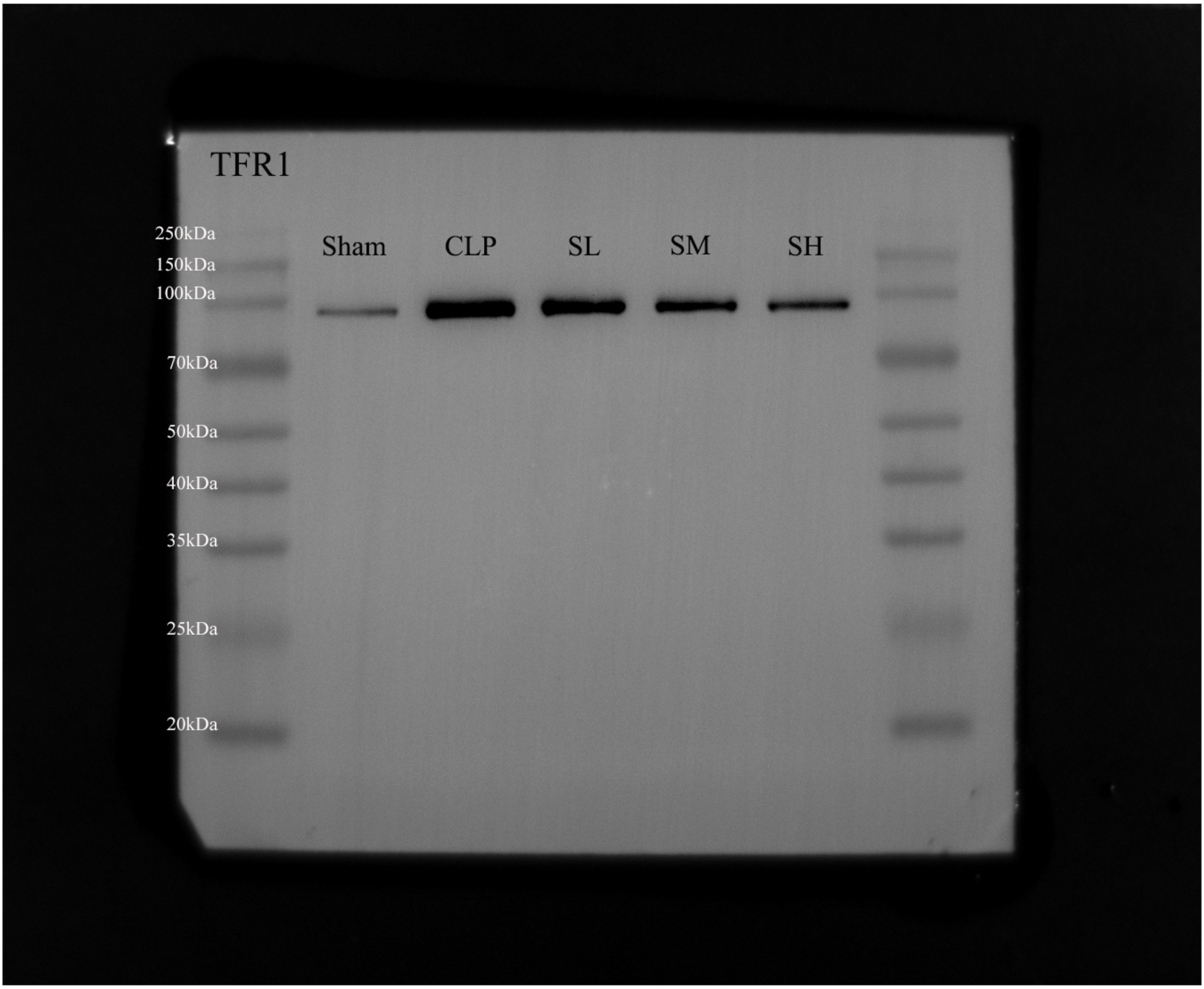


Representative Western blot images of TFR1 protein expression in lung tissue.Fig 7 was generated from this original image.

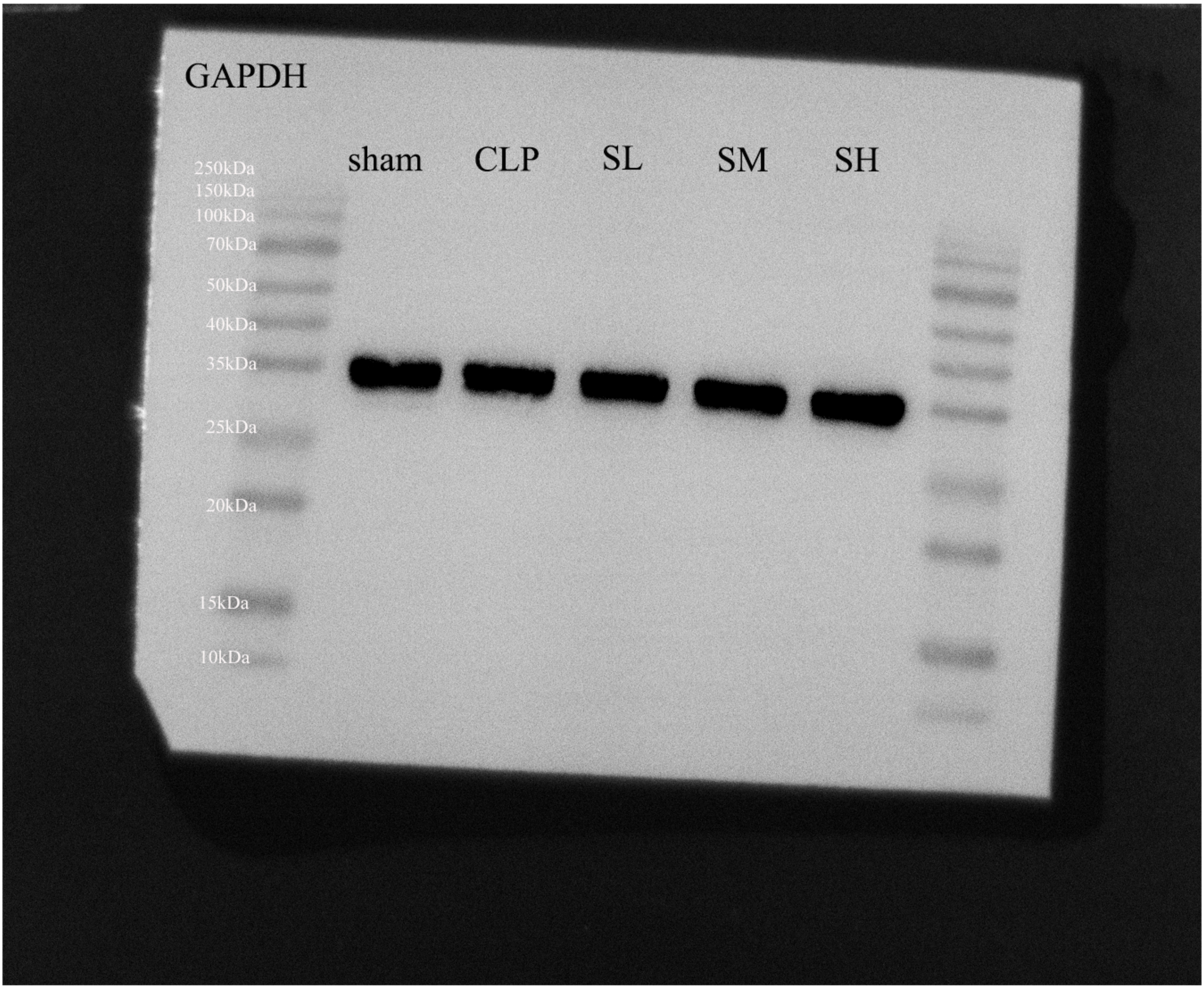


GAPDH was used as a loading control for parallel samples.Fig 7 was generated from this original image.

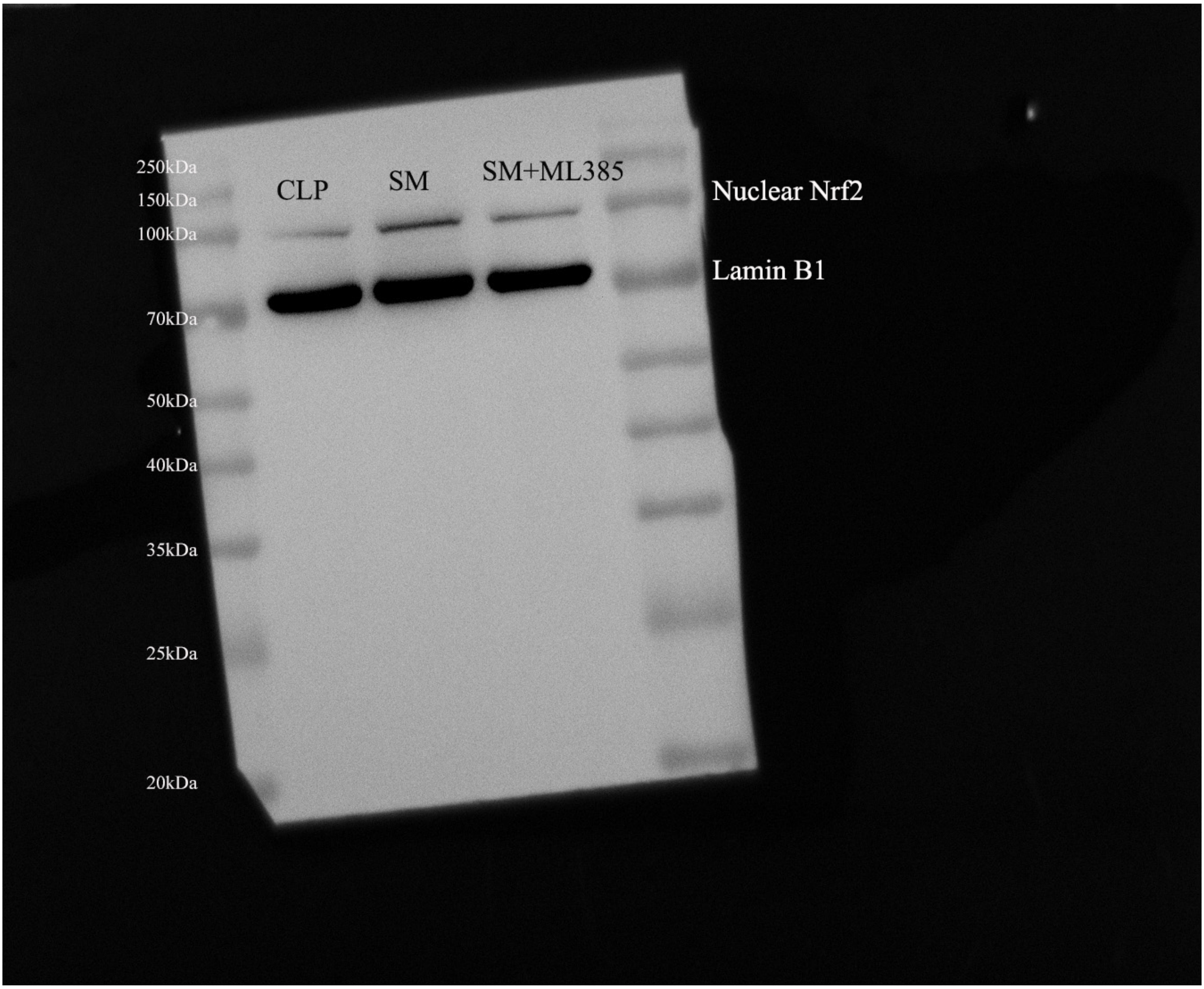


Representative Western blot images showing nuclear NRF2 protein (upper panel) and Lamin B1 (lower panel) in lung tissue from the indicated groups. Lamin B1 was used as the nuclear loading control.Fig 11 was generated from this original image.

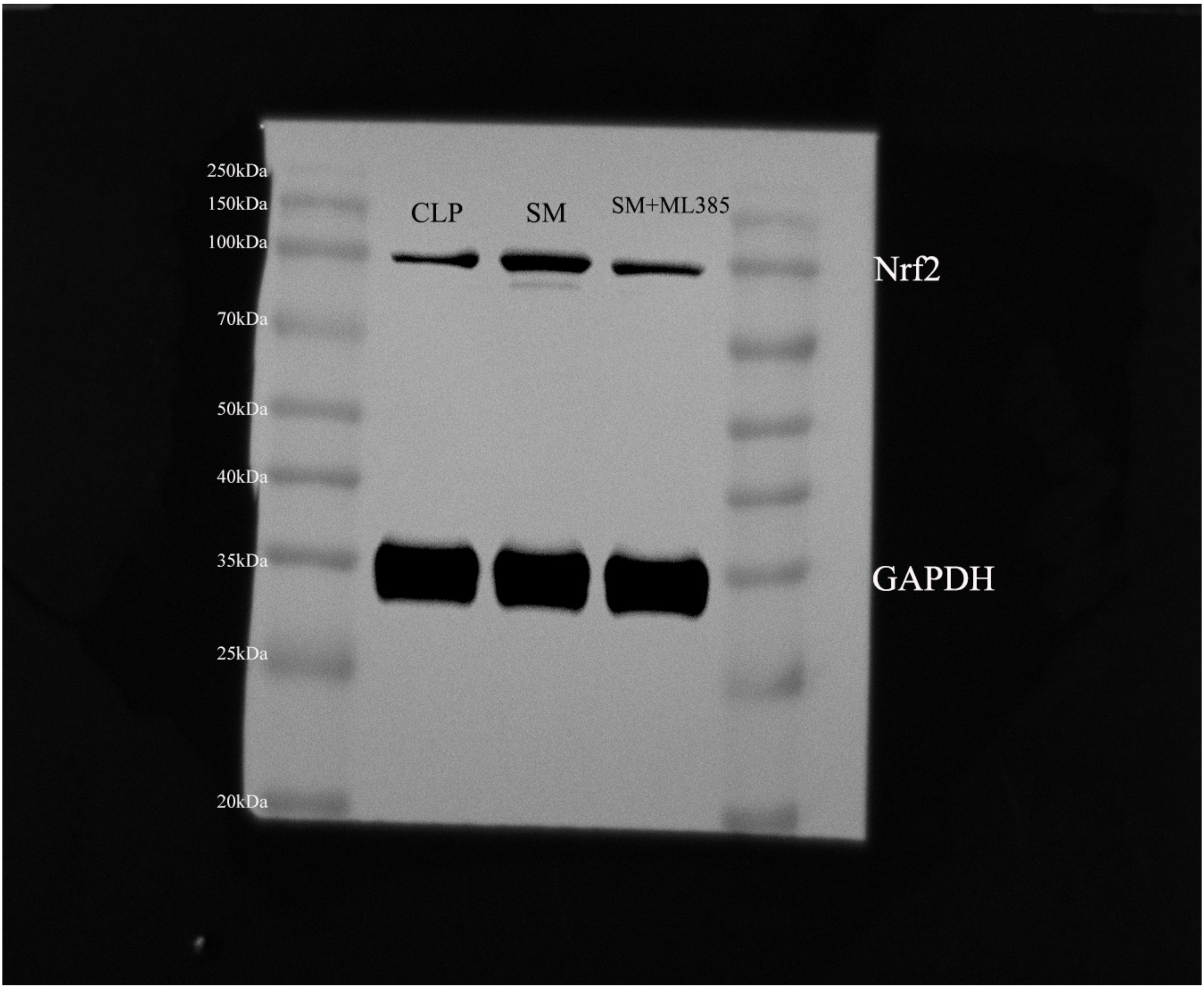


Representative original Western blot images showing NRF2 protein expression (upper panel) and GAPDH (lower panel) in lung tissue from the indicated groups. GAPDH was used as a loading control.Fig 12 was generated from this original image.

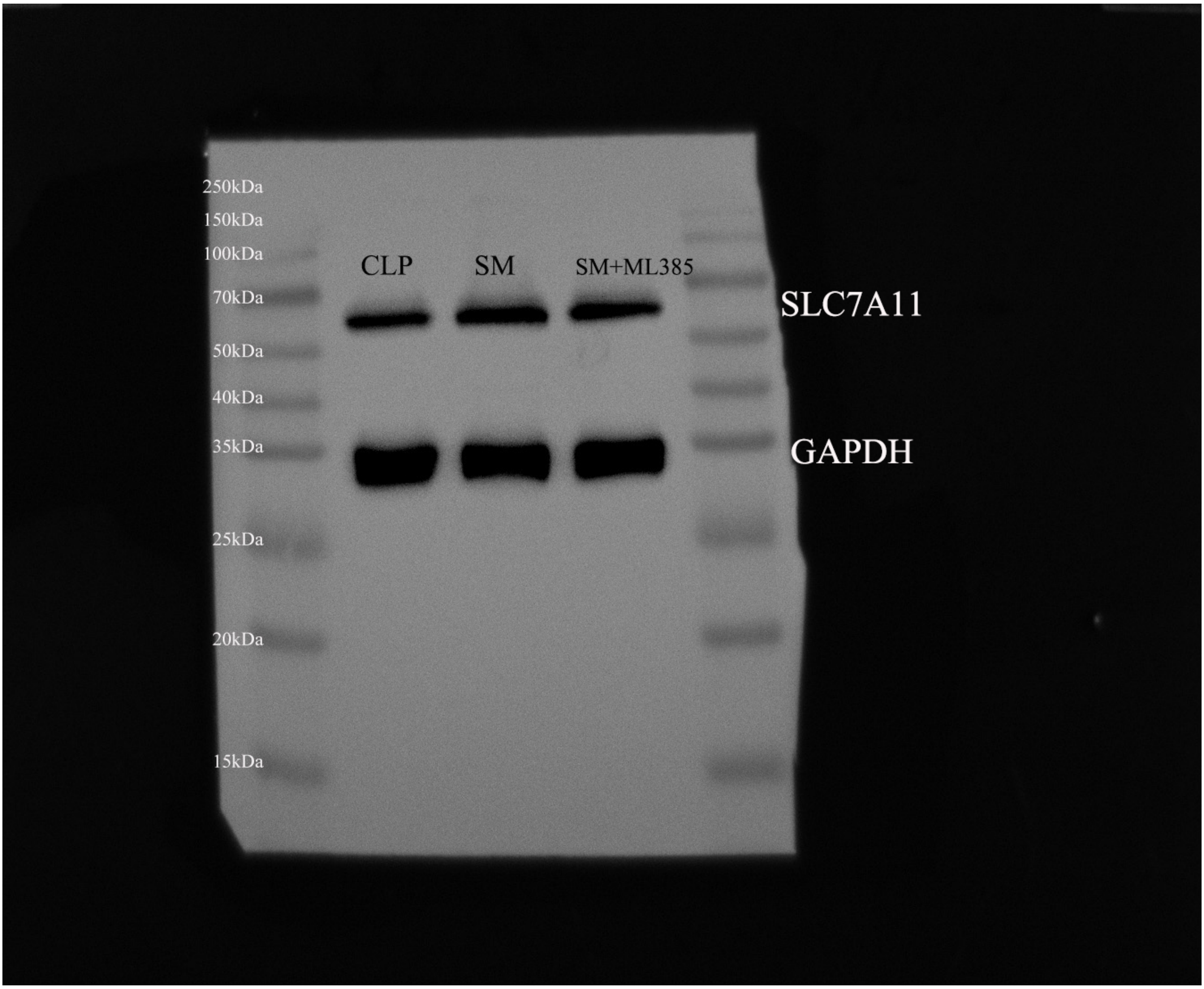


Representative original Western blot images showing SLC7A11 protein expression (upper panel) and GAPDH (lower panel) in lung tissue from the indicated groups. GAPDH was used as a loading control.Fig 12 was generated from this original image.

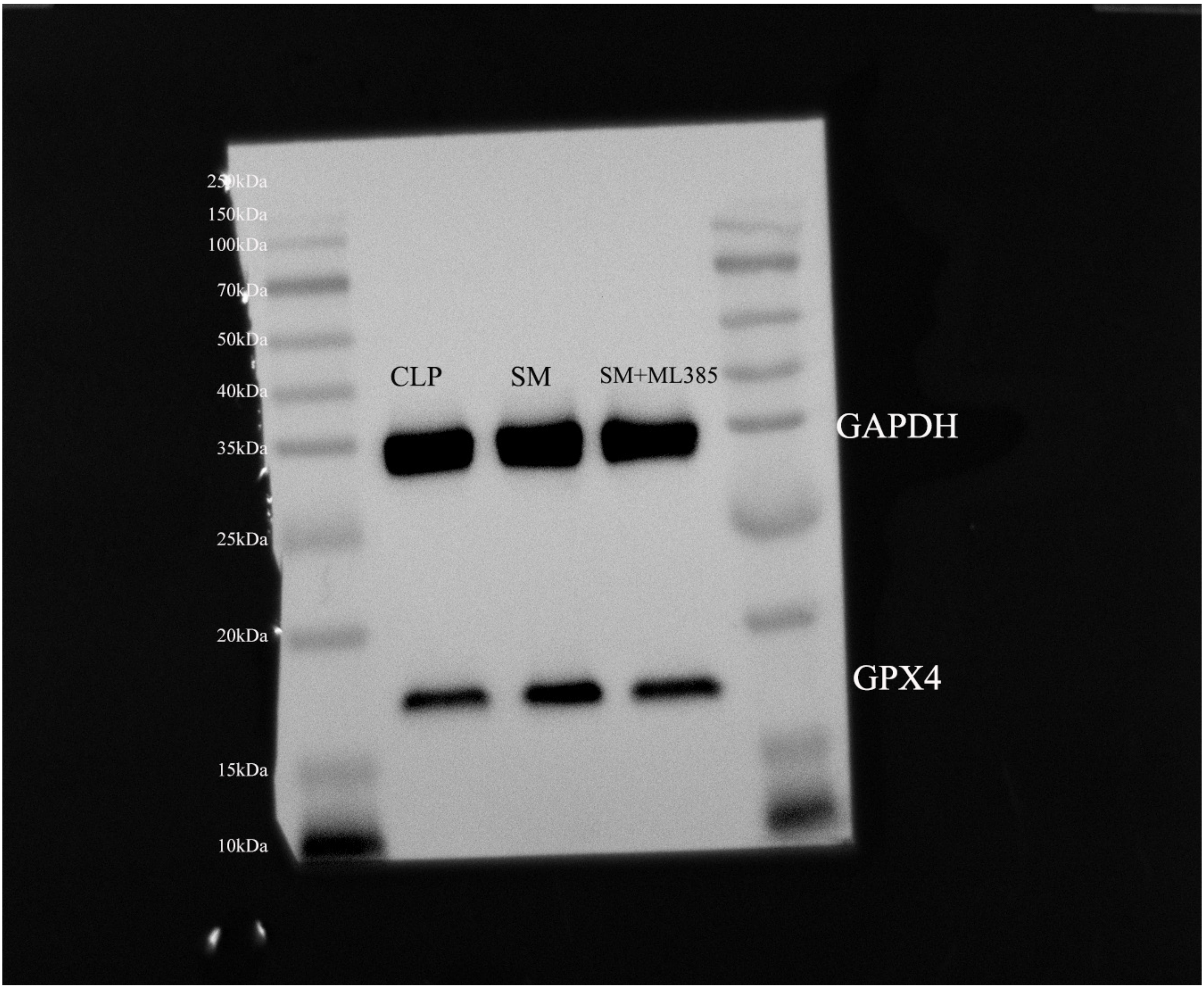


Representative original Western blot images showing GPX4 protein expression (lower panel) and GAPDH (upper panel) in lung tissue from the indicated groups. GAPDH was used as a loading control.Fig 12 was generated from this original image.

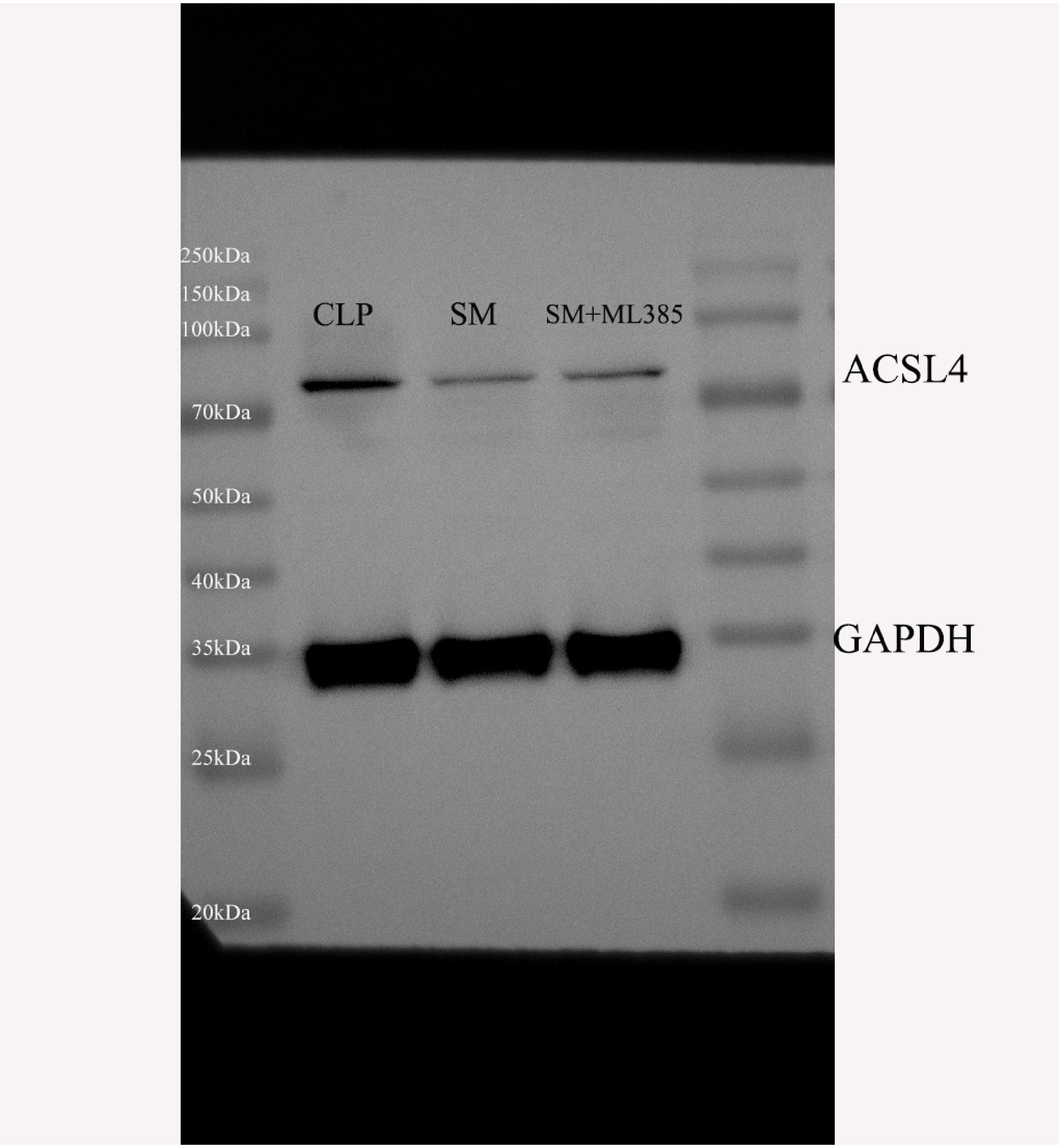


Representative original Western blot images showing ACSL 4protein expression (upper panel) and GAPDH (lower panel) in lung tissue from the indicated groups. GAPDH was used as a loading control.Fig 12 was generated from this original image.

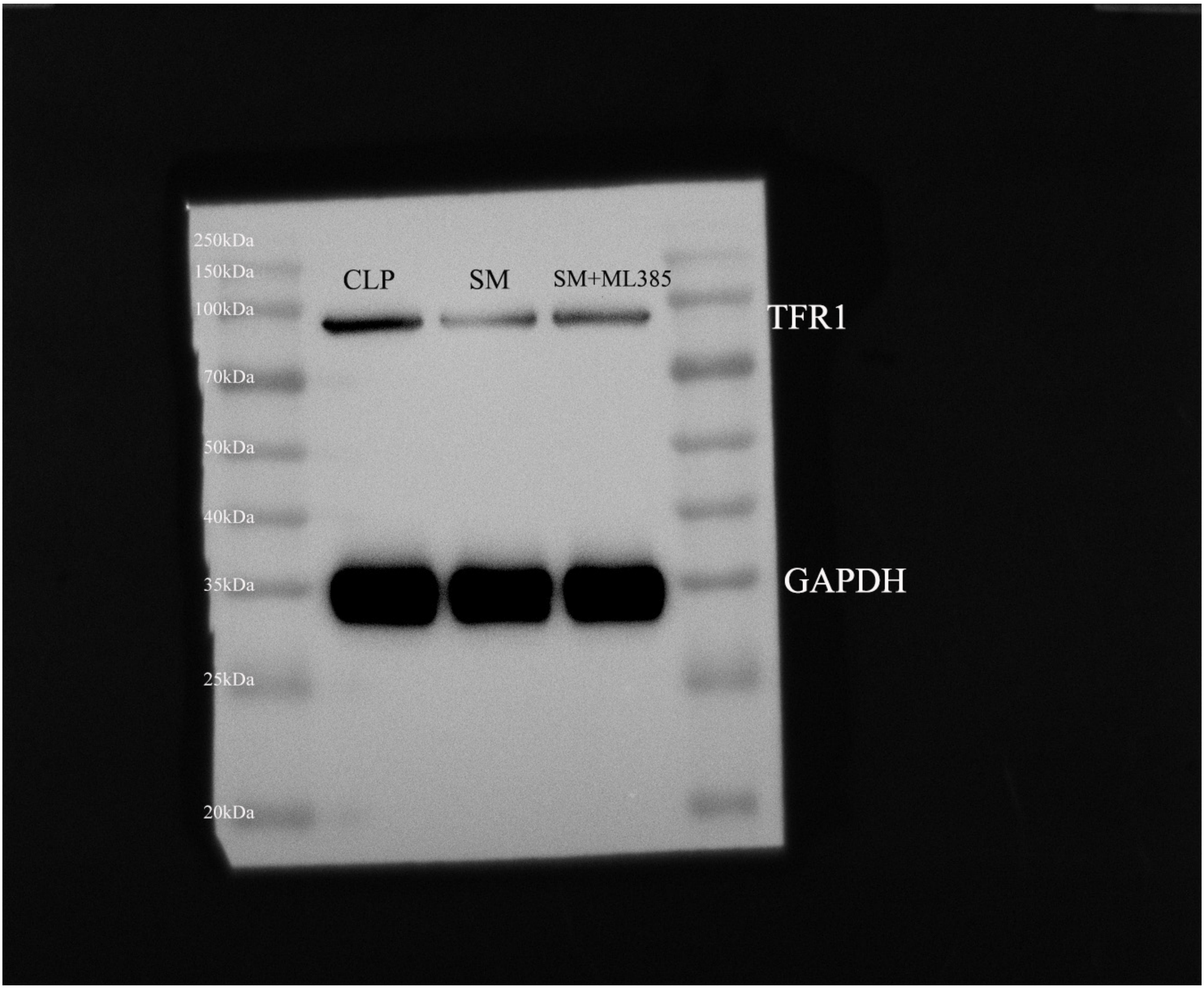


Representative original Western blot images showing TFR1protein expression (upper panel) and GAPDH (lower panel) in lung tissue from the indicated groups. GAPDH was used as a loading control.Fig 12 was generated from this original image.
